# Intestinal toxicity evaluation of polygala total saponins in mice according to toxicological evidence chain (TEC) concept

**DOI:** 10.3389/fphar.2026.1782000

**Published:** 2026-05-20

**Authors:** Yulu Liang, Yihong Li, Jiaqi Xie, Yuhan Sun, Kaikai Liu, Junbo Cui, Xingyu Gao, Chuanxin Liu, Jianmei Huang

**Affiliations:** 1 School of Chinese Materia Medica, Beijing University of Chinese Medicine, Beijing, China; 2 Henan Key Laboratory of Rare Diseases, Endocrinology and Metabolism Center, The First Affiliated Hospital, and College of Clinical Medicine of Henan University of Science and Technology, Luoyang, China

**Keywords:** arachidonic acid metabolism, intestinal microbiota, intestinal toxicity, polygala total saponins, toxicological evidence chain

## Abstract

**Background:**

Polygalae Radix a widely used tranquilizing traditional Chinese medicine, is limited by gastrointestinal adverse effects. The Polygala total saponins are identified as its major toxic constituents.

**Methods:**

To clarify the toxicity of PTS and elucidate its underlying toxic mechanism, this study performed five key experiments: First, we observed the toxic manifestations of PTS in male ICR mice and collected serum and intestinal tissue samples after administration. Second, we obtained toxicological evidence through histopathological examination and serum biochemical analysis. Third, we detected the protein and mRNA expression levels of tight junctions (TJs) in intestinal tissues, as well as the content of short-chain fatty acids (SCFAs) in feces. Fourth, we analyzed fecal microbial community structure using 16S rRNA gene sequencing after PTS exposure. Fifth, we explored the potential toxic mechanisms using metabolomics and transcriptomic analysis.

**Results:**

Three days after PTS administration (i.g., 120 and 240 mg/kg/d), mice exhibited obvious intestinal damage. The main mechanisms underlying PTS-induced intestinal toxicity involved disrupted digestive and absorptive functions, enhanced intestinal inflammation, impaired intestinal barrier integrity (including compromised tight junctions), and severe intestinal microbiota dysbiosis. Notably, intestinal microbiota dysbiosis was closely correlated with body weight loss and intestinal barrier injury in PTS-treated mice. Further analysis revealed that PTS significantly reduced the abundance of multiple genera within the Lachnospiraceae family and tended to decrease short-chain fatty acid (SCFA) levels. Mechanistically, PTS promoted intestinal inflammation at least partially by upregulating arachidonic acid metabolism.

**Conclusion:**

This study interprets the mechanism of intestinal toxicity of PTS from the perspectives of metabolites, DEGs, and microbiota. This helps us refine the toxicological evidence chain (TEC) of PTS and further understand its toxicity mechanism.

## Graphical Abstract

**Figure:**
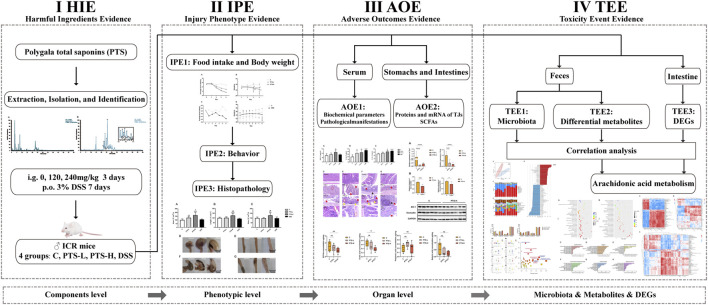

## Highlights

- The toxicological evidence chain (TEC) concept was applied to the whole process of the intestinal toxicity evaluation of Polygala total saponins (PTS).
- The key mechanism of intestinal toxicity induced by PTS (i.g., 240 mg/kg/d, 3 days) involves impaired digestion and absorption, excessive inflammatory responses, intestinal barrier disruption (including tight junction proteins), and intestinal microbiota dysbiosis, which involves a decrease in the abundance of multiple genera within the Lachnospiraceae family.
- We analyzed the toxicity of PTS from the perspective of intestinal microbiota, fecal metabolites and intestinal gene expression.
- Arachidonic acid metabolism played a crucial role in the intestinal inflammation induced by PTS, which leads to intestinal damage.

## 1 Introduction

Polygalae Radix (PR, Yuanzhi) is derived from the roots of *Polygala tenuifolia* Willd. and *Polygala sibirica* L. (Polygalaceae). It is one of the widely used traditional Chinese medicines, which was first recorded in Sheng Nong’s herbal classic. Pharmacological studies have revealed that PR possesses a variety of biological activities, such as nootropic effect, sedative-hypnotic, neuroprotection, antidepressant, expectorant, antitussive, anti-aging, diuretic, anti-inflammatory and immunomodulatory, etc. (Wang et al., 2020). However, PR can cause gastrointestinal toxicity which restricts its application. Clinically, oral intake of high doses of PR causes symptoms such as nausea, vomiting, diarrhea, and hemolysis (Zhang et al., 2023). Based on published studies, Polygala total saponins (PTS) are considered major toxic constituents in PR, and the location of the injury is mainly reflected in the gastrointestinal tract (Xin et al., 2020). Therefore, in order to make better use of PR, we need to have a deeper understanding of its toxic mechanisms.

The Toxicological Evidence Chain (TEC) framework was first proposed and applied in 2019 by our research group (Liu et al., 2020; Kong et al., 2022). To investigate the whole process of toxic drugs that began with looking for potential toxic components to obtain Harmful Ingredients Evidence (HIE), we attempted to capture Injury Phenotype Evidence (IPE, identifying toxic injury manifestations), Adverse Outcomes Evidence (AOE, confirming significant damage to target organs) and Toxicity Event Evidence (TEE, identifying key toxic targets, small molecules, and pathways). Therefore, under the framework of the TEC, this study integrated techniques including 16S RNA, metabolomics, and transcriptomics to comprehensively analyze the intestinal toxicity mechanism of PTS.

## 2 Materials and methods

### 2.1 Drugs and reagents

PR was purchased from Anguo Xingye Traditional Chinese Medicine Planting Technology Development Co., Ltd., which was identified as the dry root of *P. tenuifolia* Willd by Professor Yaojun Yang (Beijing University of Chinese Medicine, China). The mobile phases consisted of Acetonitrile (LC-MS, Thermo Fisher, United States), formic acid (LC-MS, RHAWN, Shanghai YIEN Chemical Technology Co., Ltd, China) and Methanol (LC-MS, Fisher, United States). Ultra-pure water was generated by Milli-Q Integral water purification system (Millipore, United States). Dextran sulphate sodium salt (DSS, 36,000–50000 MW, MB5535) was purchased from Dalian Meilun Biotechnology Co., Ltd. Isoflurane was purchased from Tianjin Ringpu Biotechnology Co., Ltd.

### 2.2 Extraction, isolation, and identification of PTS (HIE)

The dried and crushed PR sample (100.0 g) was extracted with 70% ethanol (1,000 mL) three times (3 h, 2 h, 2 h) at heating reflux before being filtered. The solution was concentrated under reduced pressure and then separated by methanol-activated macroporous resin D101. Subsequently, the packing column was successively eluted with water, 30%, 50%, 70%, and 95% ethanol until the eluate was clear and colorless each time. The eluents of 70% and 95% were collected and then concentrated under reduced pressure to remove the solvent. After freeze-drying, the powder of PTS was obtained.

The quantitative analysis was performed by HPLC and the qualitative analysis was performed by Q Exactive LC-MS. The contents of Tenuifolin were determined by Agilent 1,260 high-performance liquid chromatograph (HPLC, VWD detector, Agilent Technologies, United States) according to the Content Determination of PR from the Chinese Pharmacopoeia (edition 2020). Chromatographic separations were carried out on an Elite Hypersil ODS 2.5 μm 4.6 mm*150 mm (E1921153, China). The chromatographic and mass spectrometric parameters of LC-MS in this study were completely consistent with those in our previously published and validated method (Sun et al., 2026).

### 2.3 Intestinal toxicity induced by PTS (AOE)

#### 2.3.1 Preparation of solution

PTS solutions were prepared in water at concentrations of 12 mg/mL and 24 mg/mL and put in the 4 °C refrigerator for storage. The DSS was dissolved in distilled water to prepare a 3% DSS solution.

#### 2.3.2 Animal handling

Sixty ICR male mice (weighing 20–25 g) purchased from SPF (Beijing) Biotechnology Co., Ltd. The animal license number was SCXK (Beijing) 2019–0010, and the animal study was approved by Beijing University of Chinese Medicine (BUCM-2023041102–2009). The animal experiment started on 1 July 2022 and ended on 10 July 2022. All experimental methods were completed in conformity with Chinese national legislation and local guidelines.

The mice were fed in the SPF animal room (23 °C ± 2 °C, humidity of 35% ± 5%) of Beijing University of Chinese Medicine, under a diurnal 12 h light cycle, and were conventionally raised for 3 d. Mice were divided into four groups, including the control group (C), the low-dose group (PTS-L), the high-dose group (PTS-H) and the positive drug group (DSS), which were randomized using a computer based random order generator. 15 mice were set in each group. The gavage volume of the C, PTS-L, and PTS-H groups was 10 mL/kg for 3 days. The DSS group consumed distilled water containing 3% DSS *ad libitum* for 7 days, and the other groups consumed distilled water *ad libitum*. In Table 1, we display the dosage for each group. Mice were observed daily for changes in appearance and activity. Before dosing, the body weight of each mouse was noted.

**TABLE 1:** Experimental design of PTS administration.

Group	Number	Drug	Dose	Administration method	Exposure period
C	15	Water	10 mL/kg	i.g., continuous administration	3 d
PTS-L	15	PTS	120 mg/kg/d		3 d
PTS-H	15	PTS	240 mg/kg/d		3 d
DSS	15	DSS	3%	p.o., Drink freely	7 d

Mice were anesthetized with 3% isoflurane and maintained under anesthesia with 1.5% isoflurane. Then we collected blood from the heart. Mice were euthanized in accordance with the Guide and NIH guidelines using CO_2_ inhalation with a displacement rate of 30% of the chamber volume/min. And it was ensured that the mouse had no spontaneous breathing, no cardiac activity, no corneal reflex, and showed no withdrawal reflex when its toe was pinched firmly. Tissue samples were then collected for subsequent analysis.

The stomach was incised along the greater curvature and divided longitudinally into two parts. The stomach contents were gently rinsed out with saline. And the duodenum, ileum, and colon were collected. Half of each sample was immersed in 4% paraformaldehyde (Beijing Lamblide Trading Co., Ltd., China), and the other half was immediately frozen in liquid nitrogen and stored at −80 °C. The colon contents were removed and placed in a sterile cryopreservation tube, which was then immediately frozen.

For the subsequent experiments, we randomly selected qualified samples from the surviving animals in each group: 10 mice per group were used for histopathological morphological observation, and 8 mice per group with qualified serum samples were used for biochemical index determination. All statistical analyses were performed based on the final valid sample size, which had sufficient statistical power to support the conclusions.

#### 2.3.3 Examination of serum biochemical parameters and pathological manifestations

The blood samples were allowed to rest for about 30 min and centrifuged once at 3,500 rpm, 4 °C and 10 min. DAO, IL-1β, and IL-18 levels of serum were determined by ELISA. Analytical kits were used according to the manufacturer’s instructions of Jiangsu Meimian Industrial Co. Ltd. (China).

Briefly, histopathology was performed by *HE* staining for gastrointestinal tissue, including routine gradient dehydration, xylene transparency, paraffin embedding, section, hematoxylin, and eosin staining. Following staining, the sections were observed under a light microscope (magnification, ×200).

#### 2.3.4 Assessment of tight junctions (TJs) proteins expression by western blot analysis

Mouse intestinal tissues were rinsed with ice-cold phosphate-buffered saline (PBS), then homogenized in RIPA lysis buffer supplemented with PMSF, protease inhibitor cocktail, and phosphatase inhibitor cocktail at a ratio of 100:1:1:1 (v/v, G2002, G2008, G2006, G2007; Servicebio, China). Supernatant was collected, and total protein concentrations were determined via a bicinchoninic acid (BCA) assay (G2026, Servicebio, China). 5× loading buffer was added to the lysate containing 20–30 μg of protein and 70 °C for 10 min, after which proteins were separated on a sodium dodecyl sulfate–polyacrylamide gel electrophoresis gel. Proteins from the gel were transferred to the PVDF membrane (G6047, Servicebio, China) 3 h. The membrane was incubated for 30 min in the protein free rapid blocking buffer (G2052, Servicebio, China). We used the primary antibody: GAPDH (1:1,000, AC002, ABclonal), ZO-1 (1:1,000, 21773-1-AP, Proteintech), and Occludin (1:1,000, 27260-1-AP, Proteintech); the secondary antibody: Muhi-rAb™ HRP-Goat (1:5000, RGAR001, Proteintech), Anti-mouse IgG-HRP (1:5000, GB23301, Servicebio). The membrane was incubated with an appropriate primary antibody in the blocking solution. After being washed in the TBS-1% Tween buffer, the membrane was incubated in an appropriate secondary antibody and developed using the Hypersensitive ECL Chemiluminescence Kit (Femtogram, G2020; Servicebio, China) on the ChemiDoc™ MP Imaging System (Bio-rad, United States).

#### 2.3.5 RNA isolation and quantitative RT-PCR

Total RNA of mouse intestinal tissues was extracted using the FastPure® Cell/Tissue Total RNA Isolation Kit V2 (Vazyme, China) according to the manufacturer’s instructions. HiScript® III All-in-one RT SuperMix Perfect for qPCR (Vazyme, China) was used for the reverse transcription reaction to obtain Complementary DNA (cDNA). qPCR was performed with 2× HyperMB Universal SYBR Green qPCR Master Mix (Sangon Biotech, China).

Primers were: *Tjp1* forward: GAT​GAG​CGG​GCT​ACC​TTA​CTG​A; reverse: GGT​TTA​GAC​ATT​CGC​TCT​TCC​TC; *Ocln* forward: GCT​GTG​ATG​TGT​GTG​AGC​TG; reverse: GAC​GGT​CTA​CCT​GGA​GGA​AC; *Gapdh* forward: CCT​CGT​CCC​GTA​GAC​AAA​ATG; reverse: TGA​GGT​CAA​TGA​AGG​GGT​CGT.

#### 2.3.6 Detection of SCFAs (short-chain fatty acids)

Add water to the feces and vortex-mix for 10 s. Homogenize (40 Hz) the fecal sample for 4 min, followed by ultrasound treatment for 5 min while maintaining it in ice water. Repeat this ultrasound step 3 times. Centrifuge the mixture at 5000 rpm and 4 °C for 20 min. Take 0.8 mL of the supernatant and add 0.1 mL of 50% sulfuric acid and 0.8 mL of an extracting solution (a 25 mg/L stock solution in methyl tert-butyl ether, used as an internal standard). Vortex-mix for 10 s, oscillate for 10 min, then perform ultrasound treatment for 10 min in ice water. Centrifuge again at 10,000 rpm and 4 °C for 15 min. Store the sample at −20 °C for 30 min. Finally, collect the supernatant for GC-MS analysis.

An Agilent GC7890B-5977B gas chromatography-mass spectrometer was employed. The system made use of an HP-FFAP capillary column. A 1 μL portion of the analyte was injected in split mode with a ratio of 10:1. Helium served as the carrier gas, with a front inlet purge flow of 3 mL/min and a gas flow rate through the column of 1.2 mL/min. The initial temperature was maintained at 50 °C for 1 min, then increased to 150 °C at a rate of 50 °C/min for 1 min, followed by a rise to 170 °C at 10 °C/min for 0 min, then to 225 °C at 25 °C/min for 1 min, and finally to 240 °C at 40 °C/min for 1 min. The temperatures for the injection port, transfer line, quadrupole, and ion source were set at 220 °C, 240 °C, 150 °C, and 240 °C respectively. In electron impact mode, the energy was −70 eV.

### 2.4 16S rRNA sequencing (TEE)

Microbial DNA was extracted from the feces of mice (12 samples) using the Stool DNA Kit (D4015-02, OMEGA, United States) according to the manufacturer’s protocols. The V3–V4 region of 16 S rDNA was amplified by PCR, which used the upstream primer 341F (CCTAYGGGRBGCASCAG) and downstream primer 806 R (GGACTACNNGGGTATCTAAT). PCR reactions, containing 4 μL of 5 × FastPfu Buffer, 2 μL of 2.5 mM dNTPs, 0.8 μL of each primer (5 μM), 0.4 μL of FastPfu Polymerase, 10 ng of template DNA, and ddH_2_O was added to raise the volume to 20 μL, were performed in triplicate. Amplicons were extracted from 2% agarose gels and purified by using the AxyPrep DNA Gel Extraction Kit (Axygen, AP-GX-250G, United States) according to the manufacturer’s specifications. The amplification system was first denatured at 95 °C for 2 min, followed by 25 cycles, including denaturation at 95 °C for 30 s, annealing at 55 °C for 30 s, extension at 72 °C for 30 s, and final extension at 72 °C for 5 min. Amplicons were purified using the AxyPrep DNA Gel Extraction Kit (Axygen, AP-GX-250G, United States). Then, purified PCR products were quantified by QuantiFluor™-ST blue fluorescence quantitative system (Promega Co., Ltd., United States). Every twenty-four amplicons with distinct barcodes were combined evenly. Next, the sequencing library was constructed, and computer sequencing was performed by the Illumina PE250 MiSeq platform (Shanghai BIOZERON Co., Ltd., China).

### 2.5 Metabolomics (TEE)

Accurately weigh 50 mg of fecal or colon content samples, add 800 µL of extraction solution (methanol: water = 4:1 (v:v), containing the internal standard L-2-chlorophenylalanine (0.02 mg/mL)), and grind for 6 min (−10 °C, 50 Hz). Then conduct low-temperature ultrasonic extraction for 30 min (5 °C, 40 KHz). Let the sample stand at −20 °C for 30 min. Centrifuge at 4 °C and 12,000 rpm for 15 min, transfer the supernatant, dilute it by 1 time, centrifuge again at 4 °C and 12,000 rpm for 15 min, and transfer the supernatant to an injection vial with an inner insert for instrumental analysis.

Pipette 10 μL of the supernatant from each sample, vortex for 1 min, centrifuge at 4 °C and 12,000 rpm for 15 min, and pipette the supernatant to prepare a QC sample. Prepare 6 QC samples in parallel and inject a QC sample every 10 samples.

The column temperature was 40 °C and the flow rate was 0.3 mL/min, with the injection volume of 5 μL. 0.1% formic acid aqueous solution (A) and 0.1% formic acid acetonitrile (B) were used as the mobile phases. Following were the steps taken for the gradient elution: 0–10 min, 99% to 50%A; 10–12 min, 50% to 1%A; 12–12.5 min, 1% to 99%A; 12.5–14 min, 99%A. Mass spectrometric conditions were those previously described (L et al., 2022). Afterwards, methodological investigation and data processing were carried out by referring to the methods in the literature (Yuan et al., 2023).

### 2.6 Transcriptomic analysis (TEE)

For the extracted RNA from mouse intestinal tissues, its quality was assessed using the 2,100 Bioanalyser (Agilent) and quantified by the ND-2000 (NanoDrop Technologies). Following the manufacturer’s instructions, 1 μg of high-quality RNA sample was utilized to prepare the RNA-Seq library using the Truseq™ RNA sample preparation Kit (Illumina, San Diego, CA, United States). The sequencing library was subsequently sequenced on a NovaSeq 6,000 Sequencing System (150bp*2, Shanghai BIOZERON Co., Ltd.). After adapter trimming and filtering, clean reads were obtained and mapped to the mouse reference genome (UCSC GRCm39/mm39) using the HISAT2 2.0.5 program. Differentially expressed genes (DEGs) for PTS-H mice relative to C samples were identified using the false discovery rate (FDR) ≤0.05 and the fold change ≥2. Heat maps were created to investigate the patterns of gene expression. To understand the functions of the DEGs, Gene Ontology (GO) term enrichment and Kyoto Encyclopedia of Genes and Genomes (KEGG) pathway analysis were carried out by Goatools 0.9.9 and KOBAS 3.0.

### 2.7 Data analysis

The data were analyzed using Prism 7.0 software (GraphPad, La Jolla, CA). Differences were deemed statistically significant if *P*-value was less than 5% (*P* < 0.05). The data were expressed in ‾*x* ± *s*. If the group’s data were normally distributed, were performed using one-way ANOVA. Otherwise, a non-parametric test was used. Spearman correlation was performed to analyze relationships between intestinal microbiota and DEGs.

## 3 Results

### 3.1 The composition and dosage of PTS (HIE)

The content of PR (calculated by tenuifolia) was 3.05% (g/g), and the content of PTS was 32.0% (g/g) via HPLC. Therefore, every Gram of PR medicinal materials was equivalent to 95.3 mg PTS. The dosage of PR is 10–15 g/day for adults (Tongyue and ZHAO, 2022). Subsequently, based on the yield of PTS (95.3 mg/g) and the body surface area of a mouse, the administered dose of PTS was 144.57–216.81 mg/kg. To sum up, the administered doses were 120 mg/kg and 240 mg/kg in this study.

In addition, the results of LC-MS showed that the base peak ion (BPI) chromatogram of PTS in positive and negative ion modes is presented in Supplementary Figure S1. A total of 28 ingredients were identified in the negative-ion mode and all 28 ingredients were saponins.

### 3.2 The toxic performance of PTS (IPE)

#### 3.2.1 Animal food intake and body weight

Supplementary Figure S2A,C showed the food intake in mice. Compared with the C group, the mean values of food intake in the PTS-L, PTS-H, and DSS groups were decreased. Compared with the PTS-L group, the declining trend of food intake in the PTS-H group was more obvious.

Supplementary Figure S2B,D showed the change of weight in mice. Compared with the C group, the weight of the PTS-L group (Day 3&4: *P* < 0.05) and PTS-H group (Day 3: *P* < 0.001, Day 4: *P* < 0.01) was decreased. Moreover, the weight of the DSS group decreased from Day 6.

#### 3.2.2 Animal behavior and activity observation

Moreover, the mice in the PTS group were dispirited and exhibited less activity, compared with the C group (No obvious changes were observed in the C group). They showed vertical hair, wheezing and tachypnea, abdominal distension and abdominal respiration, breathing with sound, and belching. In the DSS group, the mice experienced repeated hematochezia, diarrhea, and perianal blood.

#### 3.2.3 Histopathological change

After mice were administered PTS for 3 days, they were killed on the 4th day. When the abdominal cavity was opened, the stomach of PTS-L mice (*n* = 9) was swollen and filled with food or gas; the PTS-H (*n* = 14) showed more severe swelling compared to the PTS-L mice. Moreover, the DSS group showed no flatulence.

**FIGURE 1:**
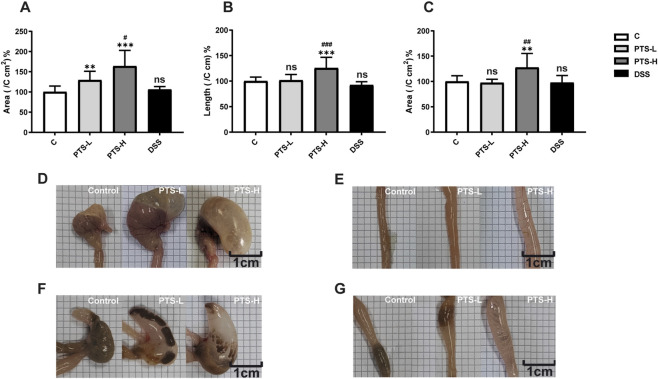
Morphological observation of gastrointestinal tissue in mice (*n* = 10). **(A)** The area of stomachs; **(B)** The diameter of duodenums; **(C)** The area of caeca. **(D–G)** The representative image of the stomach and intestine of mice **(D)** Stomach, **(E)** Duodenum, **(F)** Cecum, **(G)** Colon). Significant differences with the C group were designated as ^∗^ *P* < 0.05, ^∗∗^ *P* < 0.01, ^∗∗∗^ *P* < 0.001. Significant differences with the PTS-L group were designated as ^#^ *P* < 0.05, ^##^ *P* < 0.01, ^###^ *P* < 0.001. And ns: not significant.

The area of stomachs was significantly larger in the group treated with PTS under the naked eye (Figure 1A, PTS-L: *P* < 0.01, PTS-H: *P* < 0.001, *n* = 10). The diameter of duodenums and the area of caeca were significantly increased in PTS-H (Figure 1B, PTS-H: *P* < 0.001, *n* = 10; Figure 1C, PTS-H: *P* < 0.01, *n* = 10). In the C group, the mucosa of the gastrointestinal tract appeared smooth, pink, and elastic and mucosal folds were clear and regular. In the PTS-administration group, there was significant flatulence and hyperemia and the gastrointestinal wall became thinner (Figures 1D–G).

### 3.3 Toxicological study on intestinal toxicity induced by PTS (AOE)

#### 3.3.1 Biochemical parameters

**FIGURE 2:**
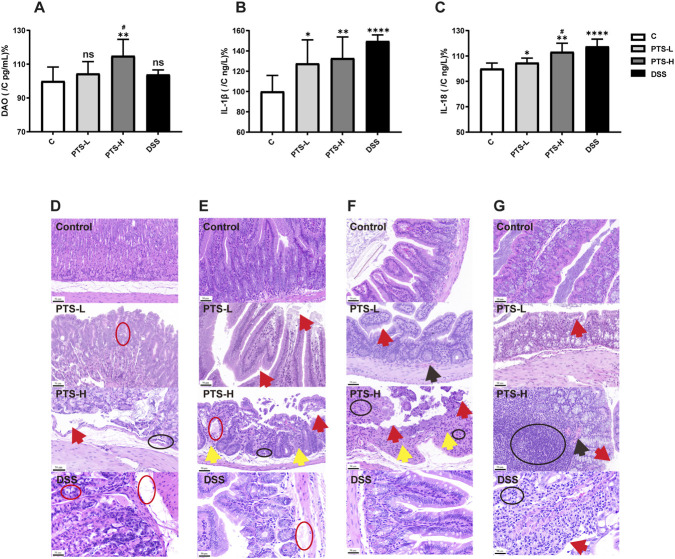
The results of serum biochemical indexes **(A)** DAO, **(B)** IL-1β, **(C)** IL-18, *n* = 8) and H&E staining **(D)** stomach, **(E)** duodenal, **(F)** ileum, **(G)** colon, ×200 magnification) in mice. Significant differences with the C group were designated as ^∗^ *P* < 0.05, ^∗∗^ *P* < 0.01, ^∗∗∗∗^ *P* < 0.0001. Significant differences with the PTS-L group were designated as ^#^ *P* < 0.05. And ns: not significant. Among them, the red circle: cellular edema; the black circle: inflammatory cell infiltration; the red arrow: epithelial cell falling off; the black arrow: hyperemia; the yellow circle: crypt disappearance.

Figures 2A–C showed the serum biochemical parameters results. The level of serum DAO (Diamine oxidase) in the PTS-H group was elevated compared to the C group (*P <* 0.01) and the PTS-L group (*P <* 0.05). However, there was no significant difference between the DSS and the C group. In addition, the level of serum IL-1β (Interleukin-1β) in the administration group was elevated compared to the C group (PTS-H: *P <* 0.01, PTS-L: *P <* 0.05, DSS: *P <* 0.0001). The level of IL-18 (Interleukin-18) in the PTS-H group was elevated compared to the C group (*P <* 0.01) and the PTS-L group (*P <* 0.05). For the PTS-L and DSS group, the level of IL-18 was elevated compared to the C group (PTS-L: *P <* 0.05, DSS: *P* < 0.0001).

#### 3.3.2 Pathological manifestations

The cell structure of each layer was clear and complete, and no inflammatory cell infiltration, vascular congestion, or edema was observed in the C group. There were minimal edema and epithelial cell shedding in the PTS-L group, but obvious tissue damage was present in the PTS-H group (Figures 2D–G). In addition, the DSS group exhibited normal or mild pathological changes in the stomach, duodenum, and ileum tissues. However, the pathological changes in colon tissues were relatively severe. Part of the glandular structure disappeared, showing exfoliated mucosal epithelial cells with the lamina propria exposed. Small amounts of inflammatory cells infiltrated.

In the stomach tissues (Figure 2D), the PTS-H group exhibited partial epithelial cell shedding and cellular edema, little lymphocyte infiltration, blood vessels that were dilated and congested, and unclear vascular endothelial cells.

In the duodenal tissues (Figure 2E), the PTS-H group exhibited partial villi shedding and the mucosa layer falling off, partial cellular edema, lymphocyte infiltration, partial crypts disappearing, blood vessels being dilated and congested, and unclear vascular endothelial cells.

In the ileum tissues (Figure 2F), the PTS-H group exhibited the villi shedding or being sparse, partial shedding of epithelial cells, lymphocyte infiltration, partial crypts disappearing, blood vessels being dilated and congested, and unclear vascular endothelial cells.

In the colon tissues (Figure 2G), the PTS-H group exhibited partial epithelial cells shedding. Partial crypts were damaged and gland duct cells were poorly ordered. Lymphocytes infiltrated the mucosa massively. Partial blood vessels were dilated and significantly congested and endothelial cells were unclear.

#### 3.3.3 The expression of TJs proteins and mRNA

**FIGURE 3:**
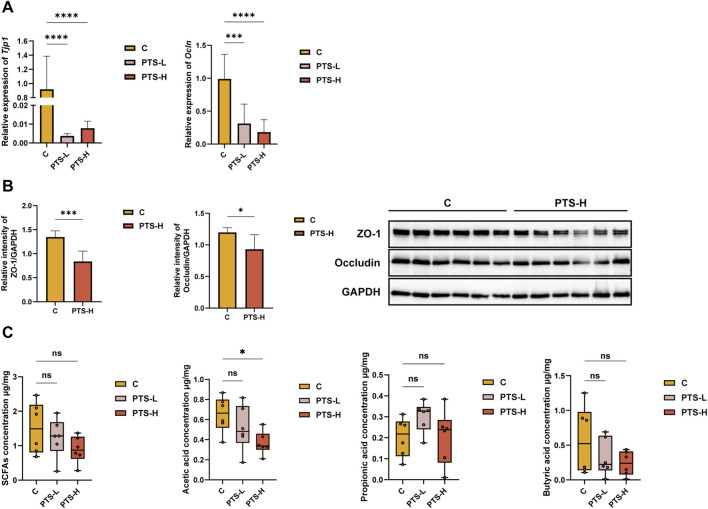
The expression of TJs mRNA (**(A)**, *n* = 8) and proteins (**(B)**, *n* = 6) in intestinal tissue and the detection results of SCFAs (**(C)**, *n* = 6) in intestinal contents. Significant differences compared with the C group are designated as: ns indicates no significant difference, ^∗^ *P* < 0.05, ^∗∗∗^ *P* < 0.001, ^∗∗∗∗^ *P* < 0.0001.

In this study, we found that the administration of PTS significantly decreased the expression of mRNA and protein of ZO-1 (mRNA: *P* < 0.0001, protein: *P* < 0.001) and Occludin (mRNA: *P* < 0.001/0.0001, protein: *P* < 0.05) in the intestine of mice (Figures 3A,B). These results suggested that PTS caused significant damage to the mechanical barrier of the mouse intestine.

#### 3.3.4 The content of SCFAs

Next, we used GC-MS to detect the content of SCFAs in the colon contents. The results are shown in Figure 3C. The total content of SCFAs, acetic acid, and butyric acid showed a decreasing trend with the increase of the PTS administration concentration. Among them, compared with the C group, the decrease in acetic acid content in the PTS-H group was significantly different (*P* < 0.05). In addition, the content of propionic acid in the PTS administration group showed a slightly upward trend, but the result was not significantly different.

### 3.4 The change of intestinal microbiota induced by PTS (TEE)

**FIGURE 4:**
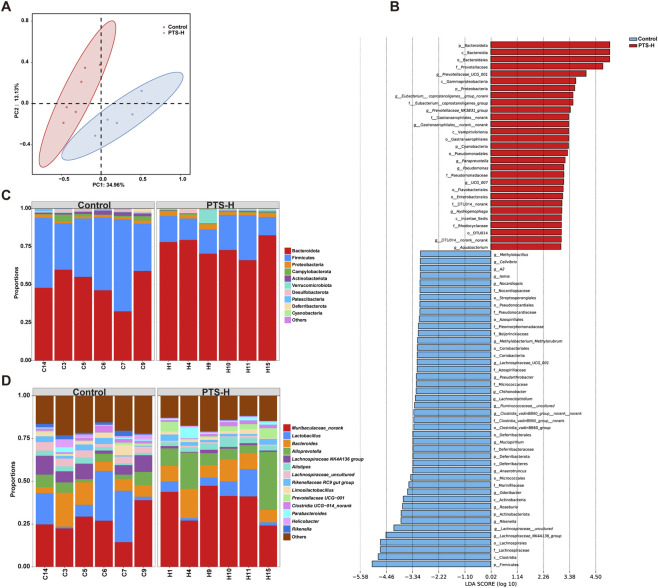
PTS-induced changes in the composition and function of intestinal microbiota. **(A)** PCoA analysis of 12 samples. **(B)** LEfSe plot of Control and PTS-H group. Identified indicator taxa grouped by filter and ranked by effect size. The threshold for LDA score was>3.0. The letter before the taxa indicates taxonomic level: “p_” for phylum; “c_” for class; “o_” for order; “f_” for family; and “*g_*” for genus. **(C)** The relative abundance of microbiota on phylum classification levels in mice. The top ten phyla in terms of relative abundance were shown in figure. **(D)** The relative abundance of microbiota on genus classification levels in mice. The genera with relative abundance >1% were presented in the figure.

Alpha diversity analysis showed that there were no significant differences in species richness and diversity between the PTS-H group and the C group (Supplementary Figure S3). PCoA analysis (Figure 4A) showed a distinct separation between the C and PTS-H groups along the first principal coordinate (34.96%), and results of ANOSIM showed that the difference between groups was significantly greater (R = 0.649, *P* = 0.005) than that within groups. It implied that PTS mainly altered the intestinal microbiota structure rather than species abundance.

The Linear Discriminant Analysis (LDA) Effect Size (LEfSe) of each component was displayed in Figure 4B (LDA score>3.0). As depicted in Figure 4C and Supplementary Figure S4, at the phylum level, PTS-H group showed significantly increased relative abundance of Bacteroidota (*P* < 0.001), Proteobacteria (*P* < 0.05) and Cyanobacteria (*P* < 0.05), and decreased Firmicutes (*P* < 0.01), Actinobacteriota (*P* < 0.01) and Deferribacterota (*P* < 0.05).

As shown in Figure 4D and Supplementary Figure S5, at the genus level, significantly downregulated genera included Lachnospiraceae*_NK4A136_group* (*P* < 0.01), Lachnospiraceae*_uncultured* (*P* < 0.001), *Rikenella* (*P* < 0.01)*, Roseburia* (*P* < 0.05), *Odoribacter* (*P* < 0.05), *Mucispirillum* (*P* < 0.05), *Lachnoclostridium* (*P* < 0.05), Lachnospiraceae *UCG_001* (*P* < 0.05) and Lachnospiraceae *A2* (*P* < 0.01); significantly upregulated genera included Prevotellaceae *UCG_001* (*P* < 0.05), *[Eubacterium] coprostanoligenes group_norank* (*P* < 0.05), Prevotellaceae*_NK3B31_group* (*P* < 0.01).

### 3.5 The change of fecal metabolites induced by PTS (TEE)

**FIGURE 5:**
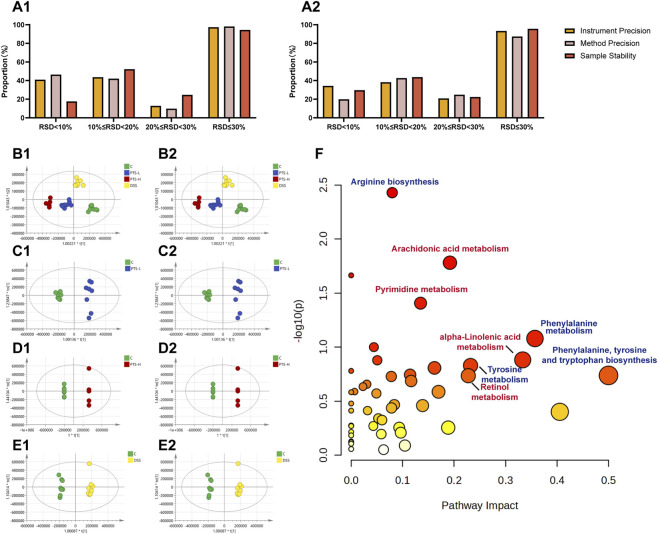
Metabolomics profile of intestinal toxicity of PTS. **(A)** Methodological investigation results; **(B–E)** Multivariate statistical analysis of fecal metabolites; **(F)** Pathway enrichment analysis of differential metabolites between C, PTS-L and PTS-H groups. 1: Positive ion mode; 2: Negative ion mode; **(B)** OPLS-DA result of 4 groups; **(C)** Control vs. PTS-L; **(D)** Control vs. PTS-H; **(E)** Control vs. DSS; **(E)** The color of the circles from white to yellow to red denotes incremental fold change (−log(p)). The size of the circles from small to large indicates an increment of the impact of pathway.

The methodological investigation confirmed that the precision and repeatability of the metabolomics detection method in this experiment were excellent (Figure 5A). The high overlap of chromatograms of QC samples indicated the stability of the detection system (Supplementary Figures S6,S7). Orthogonal partial least squares discrimination analysis (OPLS-DA) of all group samples was performed, and each group could be separated from each other (Figures 5B–E), indicating that the levels of certain metabolites in the mice changed under pathological conditions. In the OPLS-DA model, *R* ^2^ and Q^2^ were satisfied (Supplementary Table S2). The results of the permutation test indicate that the model was stable and dependable and could be used for further data analysis.

Compounds with both *VIP* > 1 and *P* < 0.05 were considered as differential metabolites. The molecular formulas of the compounds were determined according to the exact mass numbers and adduct ion forms in the peak list. The standard spectrum library or theoretical fragmentation library was retrieved from the HMDB database and compared with the secondary fragments of the original data to screen and identify the metabolites. A total of 308 differential compounds were identified, and the identification results are shown in Supplementary Table S3. The main differential metabolites related to PTS-induced intestinal toxicity included 5,7-Dihydroxyisoflavone, Tyrosol, Glycitein, maltotetraose, N-acetylhistamine, taurocholic acid (all downregulated, *P* < 0.05, Supplementary Table S3); the most significantly enriched metabolic pathway included “arachidonic acid (AA) metabolism” (Figure 5F).

### 3.6 Effects of PTS on intestinal mRNA expression profiles and genes involved in signaling pathways (TEE)

To obtain the potential mechanism of intestinal toxicity induced by PTS, we selected intestines from the PTS-H and C groups for RNA-Seq analysis. Compared with the C group, a total of 1,009 differentially expressed genes were screened in the PTS-H group (Supplementary Figures S6, S308 upregulated and 701 downregulated, FDR≤0.05, FC ≥ 2).

**FIGURE 6:**
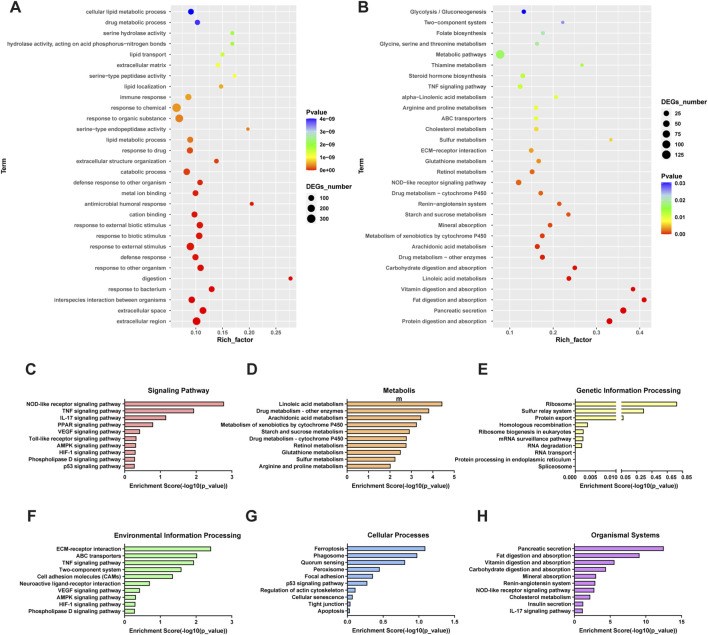
The GO and KEGG enrichment analyses between the C and PTS group. **(A)** GO terms (PTS-H vs. Control); **(B)** KEGG pathways (PTS-H vs. Control) **(C–H)** KEGG pathways (PTS-H vs. Control) of Signaling pathways **(C)**, Metabolism **(D)**, Genetic Information Processing **(E)**, Environmental Information Processing **(F)**, Cellular Processes **(G)**, and Organismal Systems **(H)** categories.

We used GO (Figure 6A) and KEGG (Figure 6B) enrichment analyses to investigate the functional properties of DEGs. According to the results of KEGG pathway enrichment analysis, DEGs participated in 217 pathways of the PTS-H group, among which 35 pathways were significantly enriched. The top significantly enriched signaling pathways were NOD-like receptor signaling pathway, TNF signaling pathway, Linoleic acid metabolism, and arachidonic acid metabolism (Figures 6C,D).

### 3.7 Association among the intestinal microbiota, DEGs and fecal metabolites (TEE)

**FIGURE 7:**
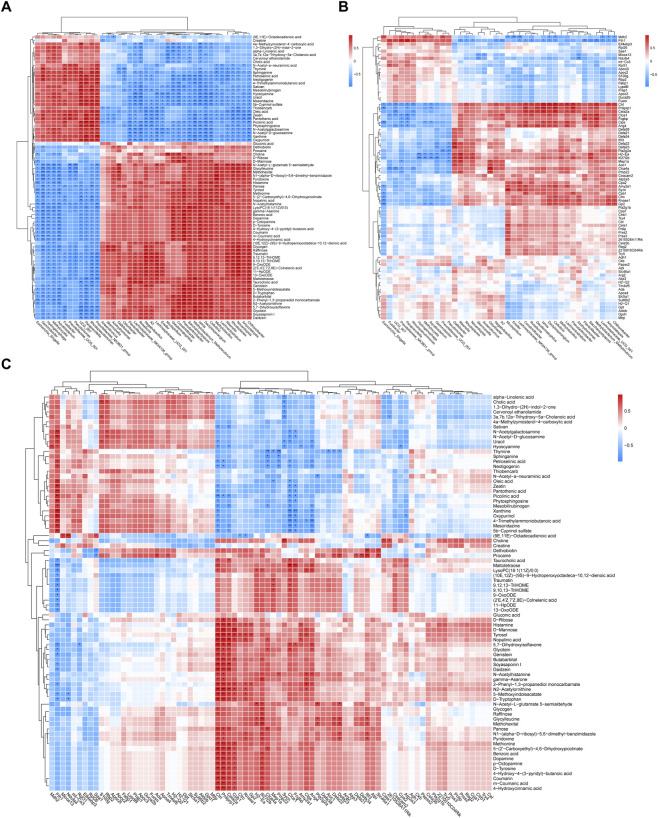
The Heatmap of the conjoint analysis between intestinal microbiota and metabolites **(A)**, intestinal microbiota and DEGs **(B)**, DEGs and metabolites **(C)**. Significant differences with Control group were designated as ^∗^ *P* < 0.05, ^∗∗^ *P* < 0.01, ^∗∗∗^ *P* < 0.001.

Spearman’s rank correlation analysis was used to evaluate the associations. These included the associations between the intestinal microbiota and the metabolites, between the intestinal microbiota and the DEGs, and between the DEGs and the metabolites. The correlation analysis results were clustered and visualized using a heatmap in Figure 7, which showed all the differential intestinal microbiota, the top 80 fecal metabolites and DEGs in terms of abundance.

The Spearman’s rank correlation coefficient was calculated for each pair of relative abundance of bacteria species and metabolites. A total of 1800 statistically significant pairs (*P* < 0.05) and 987 highly correlated pairs (|r|>0.8, *P* < 0.05) were found between the intestinal microbiota and the fecal metabolites. Similarly, 207 statistically significant pairs (*P* < 0.05) and 74 highly correlated pairs (|r|>0.8, *P* < 0.05) were found between the intestinal microbiota and the DEGs. Likewise, 354 statistically significant pairs (*P* < 0.05) and 188 highly correlated pairs (|r|>0.8, *P* < 0.05) were identified between the DEGs and the metabolites respectively. By comparing the quantity of significant pairs and the intensity of correlation, it is evident that the metabolites exhibit the strongest correlation with the intestinal microbiota.

As can be seen from Figure 7, in terms of the relationship between the intestinal microbiota and the fecal metabolites, by comparing the number of significant pairs and intensity of correlation, *Dactylosporangium*, *Saccharopolyspora* and *Iamia* were closely related to Maltotetraose; N-Acetylhistamine; Taurocholic acid. Regarding the relationship between the intestinal microbiota and the DEGs, *Ctrl*, *Ifi27l2b*, *Cela2a* and *Fth1* showed a close relationship with *Dactylosporangium* and *Cellvibrio*. Concerning the relationship between the DEGs and the metabolites, *Ctrl*, *Pnliprp1*, *Cela2a* and *Fth1* were strongly associated with 5,7-Dihydroxyisoflavone; Tyrosol and Glycitein.

*Dactylosporangium, Saccharopolyspora, Iamia* and *Cellvibrio* are associated with the digestion and degradation of carbohydrates and proteins (Lutfun and Sarker Satyajit, 2020; Shigemi, 2013; Yokota, 2014; Ákos et al., 2021; Yang et al., 2022). Secondly, maltotetraose is the main hydrolysis product of amylaceous polysaccharides (Wang et al., 2024). N-Acetylhistamine can act as a stimulant of gastric secretion (Acuña et al., 2022). Taurocholic acid, which is involved in both the emulsification and solubilization of fats during intestinal absorption, can also affect the metabolism of glucose and lipids either directly or indirectly by influencing the intestinal microbiota (Vinay et al., 2025). Additionally, the reduction of *Ctrl* leads to a weakened digestive capacity in the intestine (Sayers et al., 2021). The downregulation of these microbiota, metabolites, and genes in the PTS-H group indicates that PTS affected the digestive and absorptive functions of mice.

Apart from that, *Ifi27l2b* is related to immunity and apoptosis (Lu and Liao, 2011), and the reduction of *Ctrl* (Dóra et al., 2020) is associated with anti-inflammatory. 5,7-Dihydroxyisoflavone (refer to HMDB database, https://hmdb.ca/), Tyrosol (Guo et al., 2024) and Glycitein (Ma et al., 2024) exhibit anti-inflammatory effects. The downregulation of these metabolites and genes in the PTS-H group indicates that PTS led to intestinal inflammation. Therefore, the above results indicate that PTS led to disorders in the digestion and degradation of nutrients in the mouse intestine as well as inflammation.

## 4 Discussion

Currently, the mechanisms of toxicity induced by PR mainly include the following three aspects: Firstly, it can inhibit gastrointestinal motility and lead to the increase of the residual rate of stomachs (YANG et al., 2010); Secondly, PR affects the secretion of digestive juices, the activity of pepsin, and serum hormone levels (Cui et al., 2021); Thirdly, it can lead to gastrointestinal mucosal damage (YANG et al., 2011). Based on this, we further dissected the mechanisms of gastrointestinal damage involved in PTS toxicity from the perspective of intestinal microbiota, fecal metabolites and transcriptomics.

### 4.1 Toxic manifestations of PTS in male ICR mice

After 3 days of exposure, our study showed that the food intake and body weight in mice treated with PTS decreased significantly compared with the C group, and the longer the exposure duration, the more severe the damage, the higher the toxic dose, and the earlier the onset of toxic damage (Supplementary Figure S2). Abdominal distension and gastrointestinal flatulence were observed in the PTS-administered group.

Moreover, DAO exhibited a protective effect on the intestinal mucosa. The activity of DAO reflects the degree of damage and the integrity of the intestinal mechanical barrier. When the intestinal mucosal barrier function is impaired, intestinal villus epithelial cells will be necrotic and denuded. Subsequently, intracellular DAO will be released into the blood through the gap of damaged intestinal cells, resulting in increased activity of DAO in plasma (Shan et al., 2023). In this study, PTS caused an increase in the serum DAO (Figure 2A). It is indicated that PTS resulted in damage to the barrier in the intestinal mucosa, which increased with increasing dose. IL-1β is an important proinflammatory factor, which can initiate or boost immune responses. IL-18 directly disrupts the integrity of the epithelial cell during colitis (Roni et al., 2015). In this study, PTS caused an increase in the serum IL-1β and IL-18, which increased with increasing doses (Figures 2B,C).

It was observed before death that all mice showed wheezing and tachypnea. After death, they had serious abdominal distension and gastrointestinal flatulence (Figure 1). As for the histopathology results, PTS-induced gastrointestinal tract injury mainly led to the shedding of epithelial cells and villi, destruction of villi integrity, partial cellular edema, lymphocyte infiltration, disappearance of partial crypts, and dilation and congestion of blood vessels (Figures 2D–G).

TJs are intercellular protein complexes that maintain tissue homeostasis and integrity by regulating paracellular permeability and cell polarity. Through Western blot and quantitative RT-PCR, we found that PTS caused a significant decrease in both the protein and mRNA levels of TJs in the intestinal tissue of mice. This further demonstrates the damaging effect of PTS on the intestinal mucosa of mice.

### 4.2 PTS induced dysregulation of the intestinal microbiota in mice, especially multiple genera within the lachnospiraceae family, leading to intestinal barrier damage

To determine whether the gastrointestinal toxicity of PTS is related to the intestinal microbiota, we utilized 16S rRNA-sequencing analysis on the feces after PTS-administration. The Firmicutes/Bacteroidota ratio is associated with weight, and the decreased microbiota abundance in Firmicutes may be associated with the reduction of weight (Gao et al., 2020). Cyanobacteria are inversely related to obesity (Kaplan et al., 2019). Moreover, the abundance of *Roseburia* and *Mucispirillum* is positively correlated with weight (Nie et al., 2021; Floch, 2017). In our study, the change in abundance of these bacteria conformed to the reduction of the mouse weight. Therefore, after the administration of PTS, there was a significant correlation between the changes in intestinal microbiota and body weight.

From the perspective of intestinal barrier integrity and intestinal homeostasis, Proteobacteria can induce or exacerbate inflammation and are positively correlated with intestinal permeability (Jiang et al., 2023). In this study, the abundance of Proteobacteria was higher in the PTS-H group. At the genus level, Lachnospiraceae*_NK4A136_group*, Lachnospiraceae*_uncultured*, *Roseburia*, *Lachnoclostridium*, Lachnospiraceae *UCG_001* and Lachnospiraceae *A2* belong to Lachnospiraceae. Lachnospiraceae participate in the production of SCFAs (Meehan and Beiko, 2014), which are the major energy source for intestinal epithelial cells and maintain the epithelial barrier integrity (van de Wouw et al., 2021). The decrease of SCFAs content (Figure 3) was closely related to the downregulation of Lachnospiraceae in PTS-H group, which may be one of the mechanisms of intestinal barrier damage. *Mucispirillum* can create a positive environment for intestinal bacterial growth (Floch, 2017). Lachnospiraceae*_NK4A136_group* is significantly negatively correlated with permeability and plasma LPS levels among the previous studies (Ma et al., 2020). *Roseburia* supports intestinal homeostasis and could influence colon motility, immunological function, and anti-inflammatory function (Zohreh et al., 2017). *UCG_001* maintains the epithelial barrier integrity (Ibrahim et al., 2019), which is associated with the inhibition of colon inflammation and tumorigenesis (Yun et al., 2021). *Rikenella* can contribute to the strengthening of gut barriers (Wang et al., 2022). The lack of *Odoribacter* results in reduced SCFAs leading to inflammation (Gom et al., 2016). The abundances of the above-mentioned bacteria decreased in the PTS group, which indicated that PTS could cause gastrointestinal inflammation, the injury of intestinal barriers, and intestinal microbiota dysbiosis.

Therefore, the main effect of PTS-induced intestinal microbiota changes were reflected in the following two aspects: reducing weight, leading to inflammation, causing intestinal barrier injury, reducing the content of SCFAs, and influencing intestinal homeostasis.

### 4.3 PTS upregulated the arachidonic acid metabolism, leading to intestinal inflammation

The results of KEGG analysis in both metabolomics (Figure 5F) and transcriptomics (Figure 6B&D) focused on arachidonic acid (AA) metabolism. Cellular responses to various insults result in unusually high and/or prolonged production of pro-inflammatory eicosanoids. AA is the usual substrate for eicosanoids synthesis and the most typical eicosanoids are prostaglandin E_2_ (PGE_2_) and leukotriene B_4_ (LTB_4_). These products derived from AA can induce various inflammatory responses in multiple cell types (Fritsche, 2008). In the AA metabolism pathway, PTS was involved in the regulation of all three major branches (cyclooxygenase (COX), lipoxygenase (LOX), and cytochrome P450 (CYP450) pathways), promoting a pro-inflammatory metabolic shift that contributes to intestinal toxicity (Supplementary Figure S7). Although the release of AA was partially inhibited by the downregulation of PLA2G (the enzyme responsible for liberating AA from membrane-bound phospholipids (Wang et al., 2021)), the COX pathway was dominantly activated. Although PTGS2 (COX-2) was downregulated, the downstream enzymes involved in PGE_2_ synthesis (CBR1 and PRXL2B) were significantly upregulated, leading to the accumulation of PGE_2_ and its active metabolite 15-Keto-PGE_2_. This accumulation directly drove intestinal inflammation and barrier dysfunction. Concurrently, the CYP450 branch was activated via the upregulation of CYP2E1, CYP2B, CYP2C and EPHX2, which promoted the conversion of AA to pro-inflammatory metabolites such as 20-HETE and 11,12-EET. Additionally, the upregulation of LTB_4_ and its derivatives (12-Keto-LTB_4_, 20-COOH-LTB_4_) exacerbated immune cell infiltration and mucosal inflammation, collectively contributing to PTS-induced intestinal toxicity.

In addition, PTS-induced intestinal microbiota imbalance drives AA metabolic disorder in two key ways: First, it destroys the intestinal barrier, promotes bacterial antigen translocation, and continuously activates the inflammatory response to drive AA metabolism reprogramming; Second, it disrupts the production of microbiota-derived signaling molecules (SCFAs), which directly regulate the expression of host AA metabolism-related genes. AA metabolism can also further exacerbate intestinal inflammation and barrier damage.

In conclusion, our results demonstrate that PTS induces intestinal microbiota dysbiosis and drives the pro-inflammatory reprogramming of AA metabolism, thereby inducing/exacerbating intestinal inflammation and barrier damage.

### 4.4 Limitations and future considerations

In this study, based on the body surface area equivalent conversion method, the commonly used oral dosage of PR in clinical practice was converted into the dosage for mice. Despite certain limitations in the dosing regimen of this study, attributable to the inherent physiological disparities between species and the differences in clinical medication scenarios, the dose design complies with pre-clinical toxicology research norms. This design offers a standardized dosimetric foundation for the clinical translation of the experimental findings. And the median lethal dose (LD_50_) of PTS in mice was 1.36 ± 0.27 g/kg (Hui et al., 2005). Therefore, the dosage used in this study is far lower than the LD_50_, which complies with the ICH animal ethics guidelines and the 3R principles.

However, mortality in the PTS-H group (240 mg/kg/d) reached 20% (3/15 mice) in this study. Although this dosage was below the reported LD_50_ of PTS, sporadic deaths still occurred. This may be explained by the local gastrointestinal toxicity of PTS rather than systemic toxicity, since saponins are not fully absorbed systemically and exert prominent irritant effects locally in the gut. In addition, the foaming property of saponins may cause physical discomfort or respiratory distress, thereby leading to occasional death in mice. Therefore, the observed intestinal injury, microbiota dysbiosis, metabolite disorders, and transcriptomic changes only represent the biological characteristics of tolerant individuals, and may not fully reflect the overall toxicological outcomes of the entire exposed population.

The toxic manifestations of PTS indicate that there are differences in the gastrointestinal damage patterns between PTS and DSS. First, DSS mainly causes local damage to the colon, which is mainly manifested as colonic inflammation. However, PTS induced diffuse damage to the entire gastrointestinal tract, including significant flatulence, epithelial cell shedding and inflammatory cell infiltration in the stomach, duodenum, ileum, cecum and colon. Second, DSS mainly caused colonic crypt disappearance, mucosal exfoliation and bloody diarrhea, with no gastrointestinal flatulence; while PTS mainly caused gastrointestinal flatulence, gastrointestinal wall thinning, and whole intestinal villi shedding, which is consistent with the clinical abdominal distension, nausea and vomiting adverse reactions of PR. DSS was selected as the positive control because DSS is a classic intestinal inflammation inducer. It can be compared with the intestinal toxicity pattern of PTS to clarify the specificity of PTS-induced intestinal toxicity.

In addition, previous studies have reported the toxicity of single polygalasaponin monomers: the LD_50_ of total polygalasaponins via p.o. administration in mice was 3.95 g/kg, which could significantly reduce the spontaneous activity of mice (Yi et al., 2010). The LD_50_ of Yuanzhi-1 (a single saponin monomer) via p.o. administration in female ICR mice was 86.5 ± 3.7 mg/kg (Zeng-liang et al., 2014). Onjisaponin B could increase intestinal tension and induce irregular contraction amplitude in male rabbits. Moreover, a comparison of the gastrointestinal irritation among Onjisaponin B, Tenuifolin and Senegenin found that the main difference among these three components was the number of glycosyl groups, and the toxicity decreased with the reduction of glycosyl number, suggesting that the number of glycosyl groups may be related to the gastrointestinal irritation of saponins (Wen et al., 2015). These findings on single monomers indicate that it is of great significance to isolate single saponin components from PTS and carry out systematic structure-toxicity-efficacy relationship studies in future work.

### 4.5 Applications and prospects of TEC

To comprehensively evaluate drug-induced toxicity, the concept of TEC has been suggested. TEC is a conceptual framework, which constructs an evidence-chain research method of ‘Harmful Ingredients Evidence (HIE)-Injury Phenotype Evidence (IPE)-Adverse Outcomes Evidence (AOE)-Toxicity Event Evidence (TEE)’, from hypothesis to experiment, from outside to inside. The three principles of TEC include holism, plasticity, reliability and standardization. Due to the differences in toxic manifestations and sites of action among different drugs, the construction of TEC should be based on their specific research processes. The relevant information on chemistry, histopathology, biology, and toxicology should be observed and validated to provide a framework for constructing a toxicological evidence chain and predicting drug toxicity. Therefore, the concept of TEC provides an ideal tool to establish a high throughput toxicity evaluation system and analyze the mechanisms of action of drugs. The reliability level to investigate the mechanism of intestinal toxicity upon toxicological evidence evaluation was shown in Supplementary Table S4.

## 5 Conclusion

Based on the TEC framework, the toxic components (PTS) were used for the study of intestinal toxicity according to the prior study (HIE), then the toxicological evidence was obtained from objective alterations including body weight, food intake and behavior changes (IPE), and histopathology (AOE); Subsequently, on the molecular mechanism layer (TEE), 16S rRNA, metabolomics and transcriptomics were used to analyze the effect of PTS. A preliminary TEC framework for intestinal injury caused by PTS was constructed.

The results showed that PTS induced intestinal toxicity by causing inflammation, influencing digestion and absorption, injuring intestinal barriers, and disrupting the intestinal microbiota. Specifically, PTS induces intestinal microbiota dysbiosis, significantly reduces the abundance of multiple genera within the Lachnospiraceae family, and tends to decrease short-chain fatty acid (SCFA) levels. Meanwhile, it drives the pro-inflammatory reprogramming of arachidonic acid (AA) metabolism, thereby inducing and exacerbating intestinal inflammation and barrier damage. Currently, our research provides a novel point of reference for the investigation of intestinal injury induced by PTS. Additionally, it offers fundamental guidelines for future research on toxicity of novel medicinal substances.

## Data Availability

The original contributions presented in the study are publicly available. This data can be found here: RNA-seq transcriptome profiling, NCBI repository with the accession number PRJNA1466507; 16S rRNA gene amplicon sequencing, NCBI repository with the accession number PRJNA1466549.

## Glossary

AA: Arachidonic acid
AOE: Adverse outcomes evidence
BP: Biological process
BPI: Base peak ions
CC: Cell component
DAO: Diamine oxidase
DEGs: Differentially Expressed Genes
DSS: Dextran sulphate sodium salt
ELISA: Enzyme linked immunosorbent assay
FDR: False discovery rate
GO: Gene Ontology
HE: Hematoxylin-eosin
HIE: Harmful ingredients evidence
HPLC: High Performance Liquid Chromatography
IECs: Intestinal epithelial cells
i.g.: Oral gavage
IL-18: Interleukin-18
**IL-1β**Interleukin-1β**IPE**: Interleukin-1β**IPE**Injury phenotype evidence
KEGG: Kyoto Encyclopedia of Genes and Genomes
**LC-MS**Liquid Chromatography-Mass Spectrometry**LD**_**50**_: Liquid Chromatography-Mass Spectrometry**LD**_**50**_Median lethal dose
LDA: Linear Discriminant Analysis
MF: Molecular function
OTU: Operational Taxonomic Units
**p.o.**Oral administration**PCA**: Oral administration**PCA**Principal component analysis
PCoA: Principal coordinates analysis
PR: Polygalae Radix
PTS: Polygala total saponins
SCFAs: Short chain fatty acids
TEC: Toxicological evidence chain
TEE: Toxicity event evidence
TJs: Tight junctions
vs: Versus
