# Supplementary material for: Intestinal toxicity evaluation of polygala total saponins in mice according to toxicological evidence chain (TEC) concept
Source: Front Pharmacol. 2026 May 20;17:1782000. doi: 10.3389/fphar.2026.1782000 (PMC13229883; doi:10.3389/fphar.2026.1782000)
Supplement: Supplementary file 1 [file Supplementaryfile1.docx]

## Supplementary Material

### Supplemental Figures


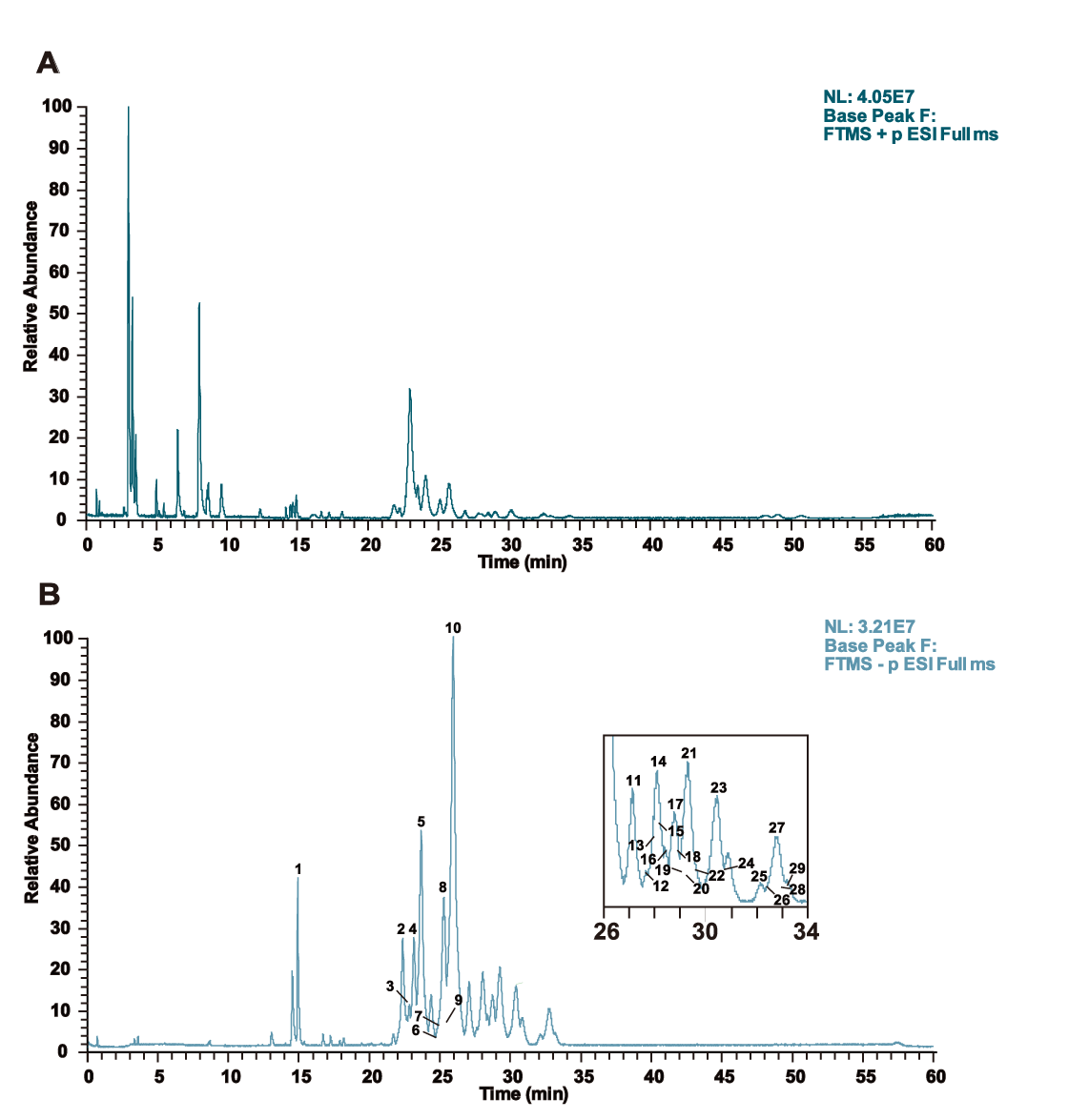


Fig.S 1 The base peak ion (BPI) of the positive ion mode (A) and negative ion mode (B) of PTS. The number in picture corresponds to the number in Tab.S1.


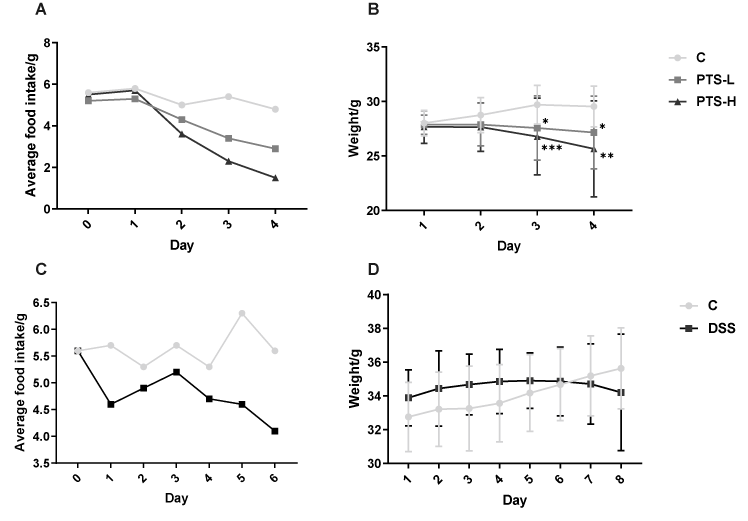


Fig.S 2 The changes in average food intake (A&C) and body weight (B&D) in mice (*n*= 9-15). Significant differences with the C group were designated as ^∗^*P* < 0.05, ^∗∗^*P* < 0.01, ^∗∗∗^*P* < 0.001.


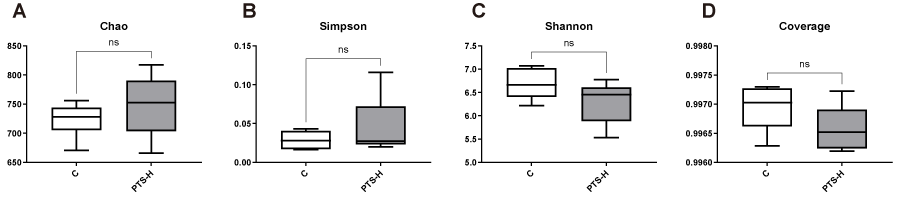


Fig.S 3 The α-diversity represented by the Chao (A), Simpson (B), Shannon (C) and coverage (D) index. Significant differences compared with the C group are designated as: ns indicates no significant difference.


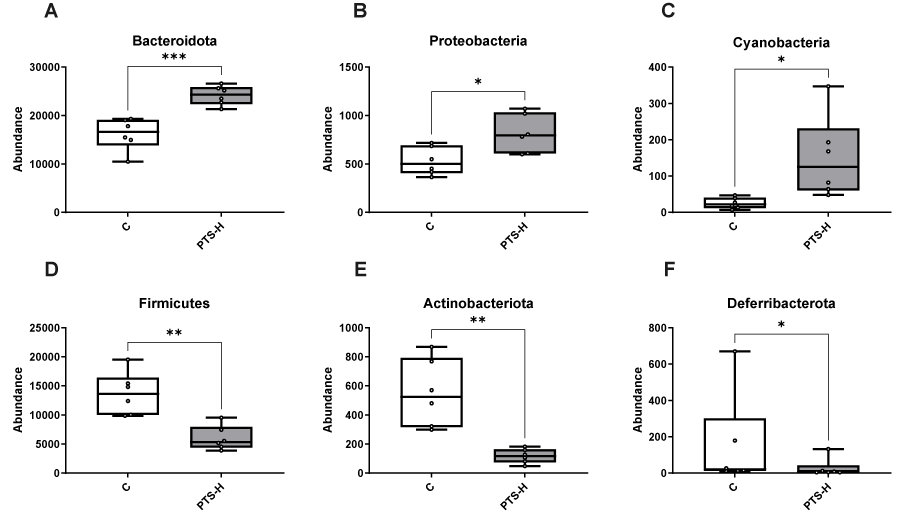


Fig.S 4 The relative abundance of microbiota on phylum level in mice. Significant differences compared with the C group are designated as: ns indicates no significant difference, ^∗^*P* < 0.05, ^∗∗^*P* < 0.01, ^∗∗∗^*P* < 0.001.


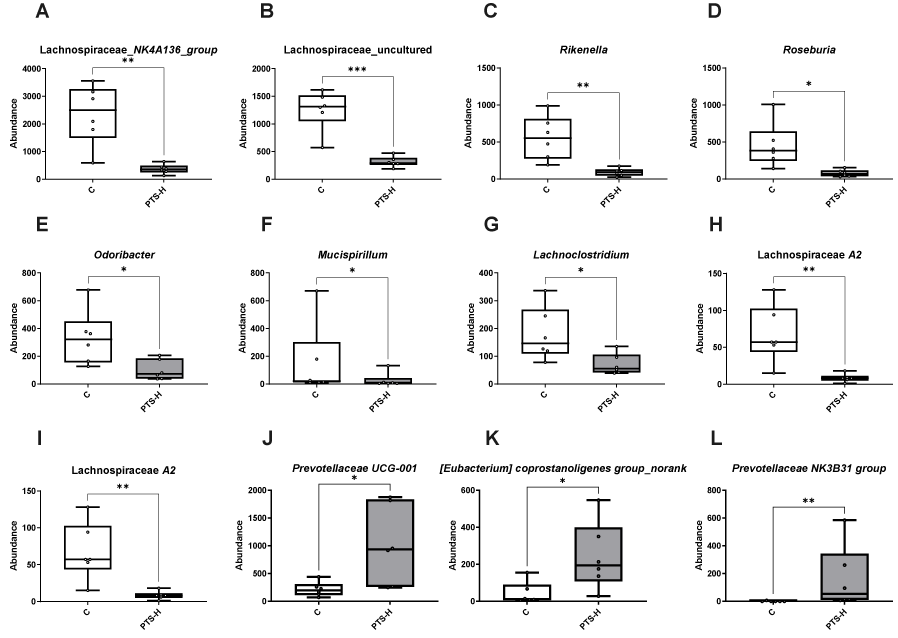


Fig.S 5 The relative abundance of microbiota on genus level in mice. Significant differences compared with the C group are designated as: ns indicates no significant difference, ^∗^*P* < 0.05, ^∗∗^*P* < 0.01, ^∗∗∗^*P* < 0.001.


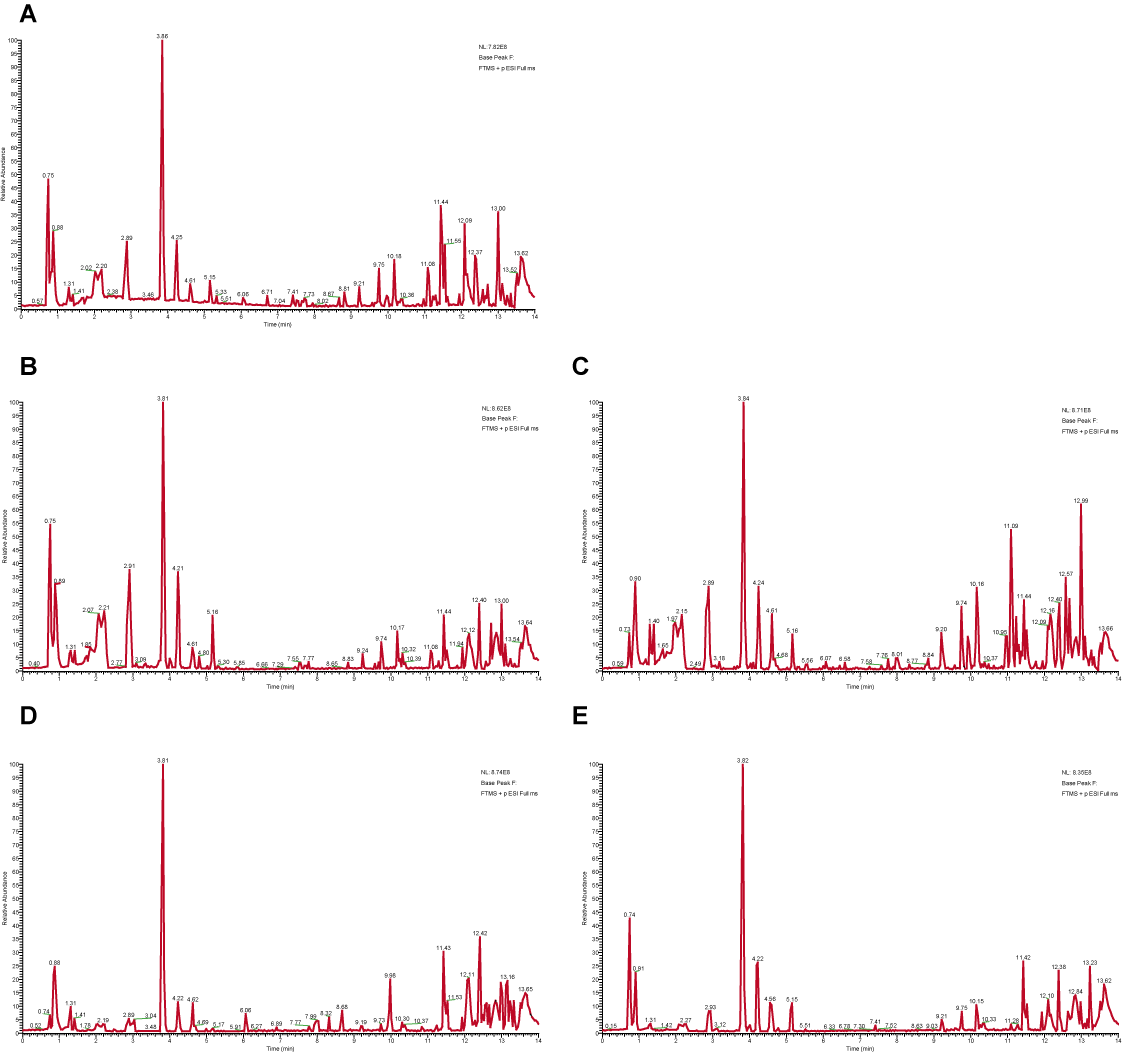


Fig.S 6 BPI chromatogram of fecal samples (positive ion mode). A: QC samples; B: Control group; C: PTS-L group; D: PTS-H group; E: DSS group


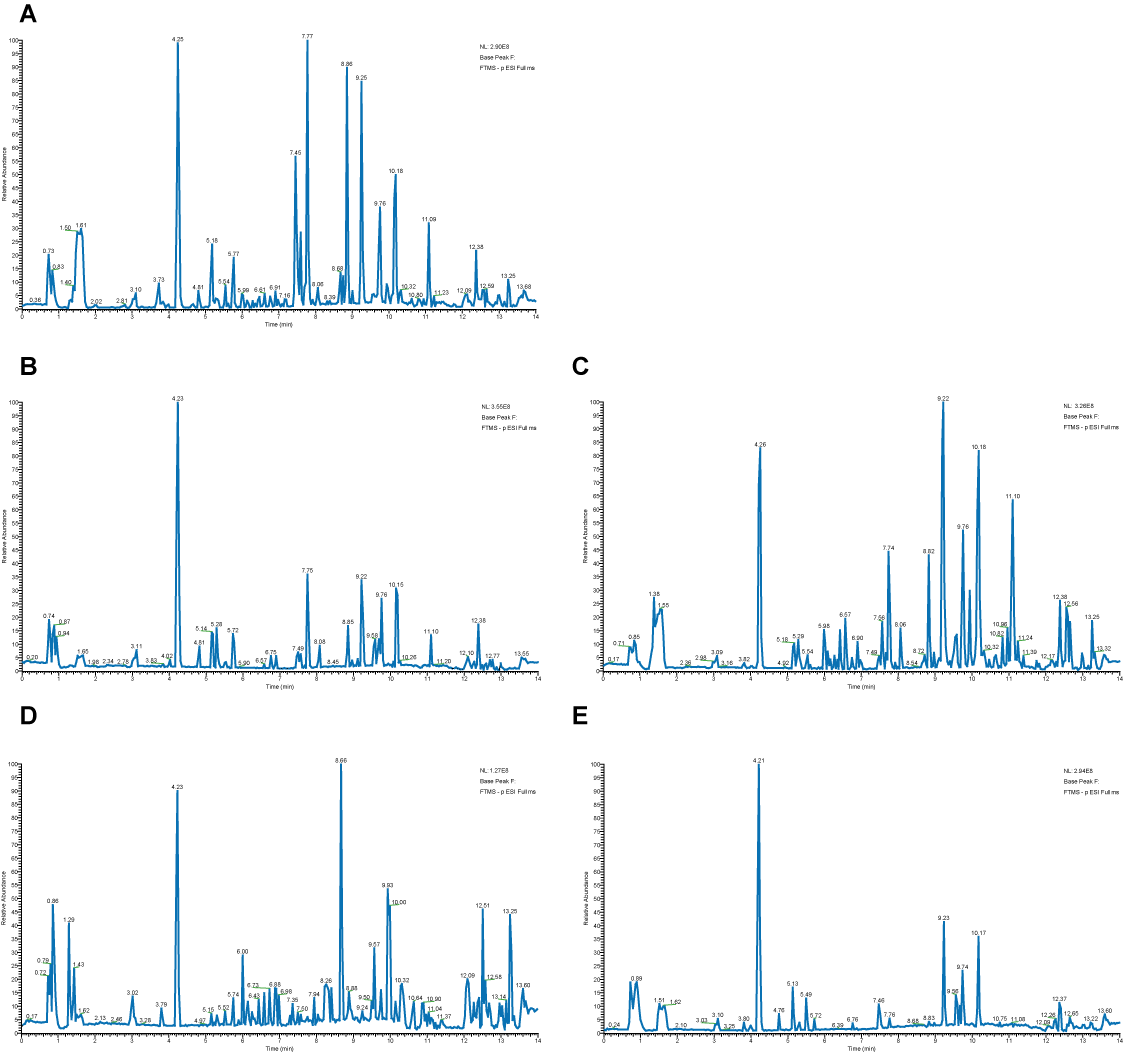


Fig.S 7 BPI chromatogram of fecal samples (negative ion mode). A: QC samples; B: Control group; C: PTS-L group; D: PTS-H group; E: DSS group


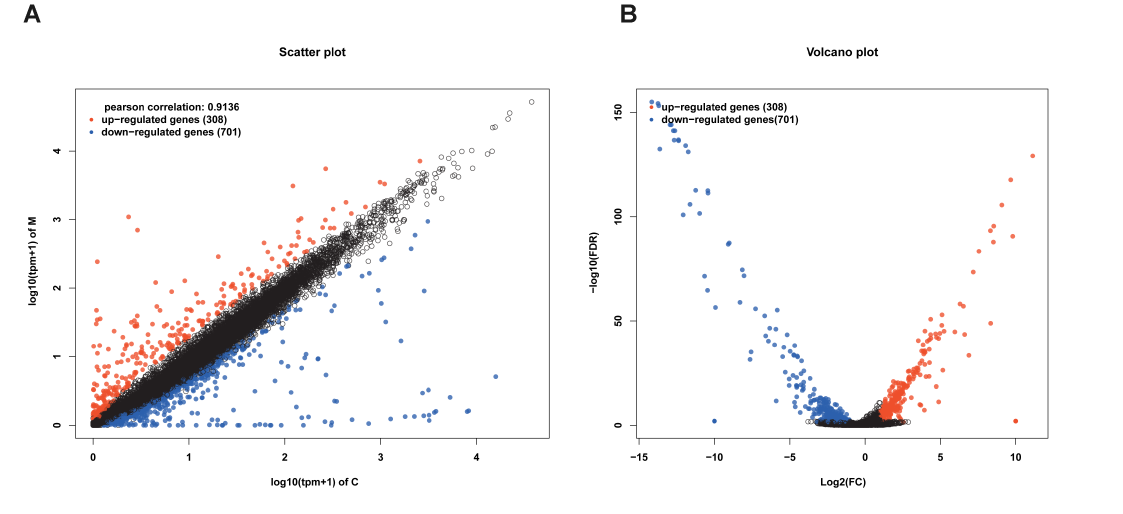


Fig.S 8 Differential mRNA expression levels for the transcriptional level of the C and PTS-H group. The Scatter-plot (A) and Volcano-plot (B) of DEGs.


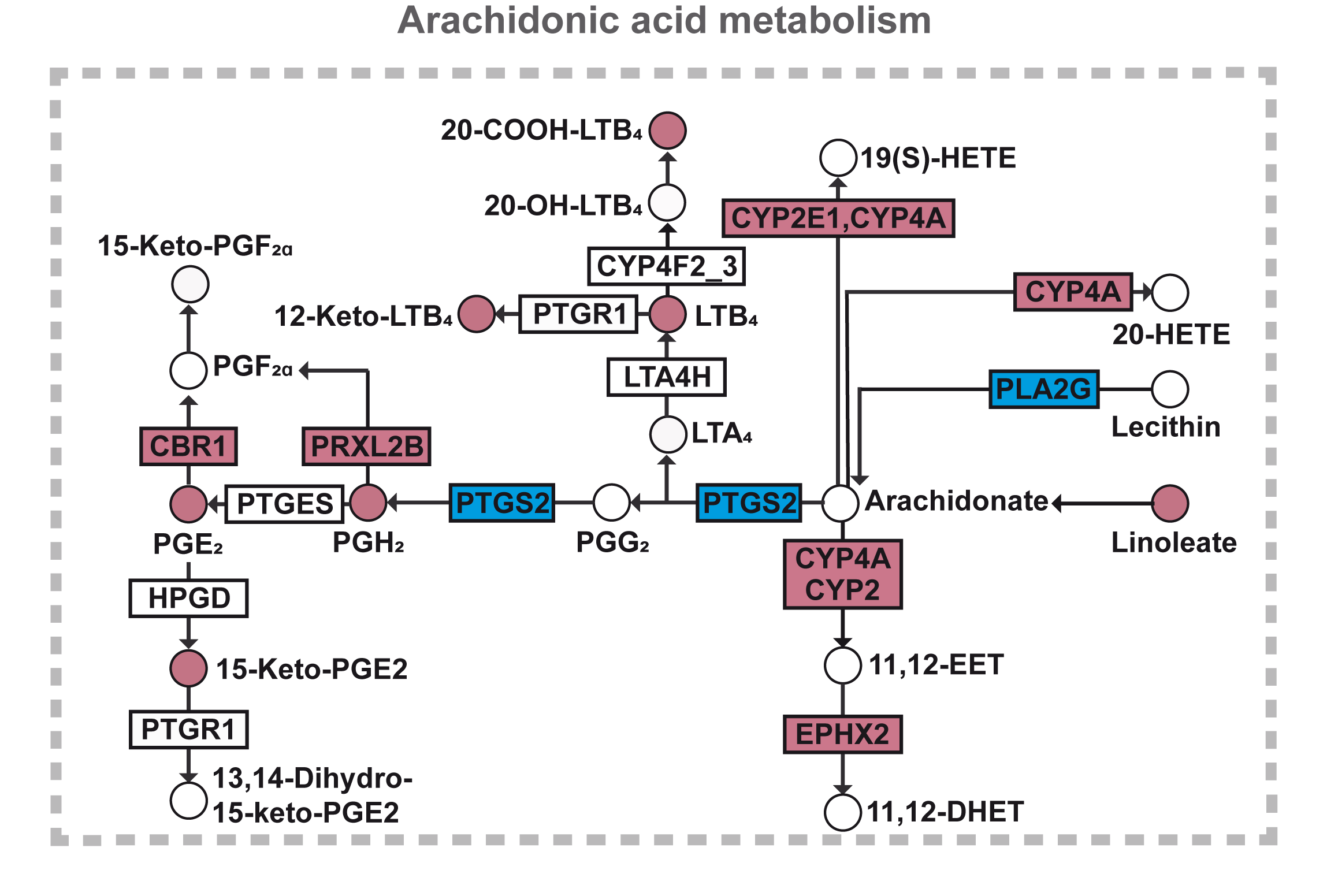


Fig.S 9 The KEGG metabolic pathway of arachidonic acid metabolism

(Red: up-regulation, blue: down-regulation. Square: gene product, mostly protein but including RNA. Circle: chemical compound.)

### Supplemental Tables

Tab. S1 The retention times, mass spectrometric information and plausible identities of the components detected from PTS.

| No. | Compounds | Molecular formula | t_R_(min) | Calculated mass (m/z) | Measured mass (m/z) | Mass error(ppm) | Major fragments ions  (m/z) | Ref. |
| --- | --- | --- | --- | --- | --- | --- | --- | --- |
| 1 | Tenuifolin | C_36_H_56_O_12_ | 14.96 | 679.36990 | 679.37219 | 3.37 | 455.31793，425.30600 | ^[1]^ |
| 2 | Onjisaponin A | C_80_H_120_O_39_ | 22.39 | 1703.73340 | 1703.73157 | 1.07 | 567.19470，455.31683，425.30630 | ^[2]^ |
| 3 | Onjisaponin WG | C_75_H_112_O_36_ | 22.89 | 1587.68605 | 1587.68201 | 2.55 | 567.19263，455.31564，425.30600 | ^[2-4]^ |
| 4 | Onjisaponin L | C_86_H_128_O_43_ | 23.34 | 1847.77566 | 1847.76648 | 4.97 | 669.23566，567.19238，455.31738，425.30618 | ^[2-5]^ |
| 5 | Onjisaponin O | C_77_H_116_O_37_ | 23.79 | 1631.71227 | 1631.70435 | 4.85 | 567.19385，455.31613，425.30597 | ^[2-4]^ |
| 6 | Onjisaponin Ug/X | C_87_H_130_O_45_ | 24.64 | 1893.78113 | 1893.77197 | 4.84 | 669.22485，537.17993，425.30655 | ^[3, 6]^ |
| 7 | Onjisaponin S | C_81_H_122_O_40_ | 24.96 | 1733.74396 | 1733.73535 | 4.97 | 669.21820，537.18353，455.31519，425.30637 | ^[4]^ |
| 8 | Onjisaponin R | C_76_H_114_O_37_ | 25.32 | 1617.69662 | 1617.68982 | 4.20 | 699.23883，455.31717，425.30606 | ^[2-4]^ |
| 9 | Senegin II | C_70_H_104_O_32_ | 25.83 | 1455.64379 | 1455.64526 | 1.01 | 1231.00769，455.32019，425.30499 | ^[4]^ |
| 10 | Onjisaponin B  (Senegin III) | C_75_H_112_O_35_ | 25.97 | 1571.69114 | 1571.68640 | 3.02 | 567.19299，455.31631，425.30627 | ^[3, 7, 8]^ |
| 11 | Onjisaponin V/Vg | C_82_H_122_O_41_ | 27.10 | 1761.73888 | 1761.73572 | 1.79 | 1617.66309，455.31671，425.30658，237.07687 | ^[3-5]^ |
| 12 | Onjisaponin H | C_74_H_110_O_34_ | 27.65 | 1541.68057 | 1541.68079 | 0.14 | 455.30682，425.30682，177.05568 | ^[2, 3]^ |
| 13 | Polygalasaponin XLV | C_78_H_116_O_38_ | 28.08 | 1659.70718 | 1659.71094 | 2.27 | 918.42529，455.31818，425.30719，237.07721，127.04002 | ^[9]^ |
| 14 | Polygalasaponin XXXII | C_79_H_118_O_38_ | 28.08 | 1673.72283 | 1673.72437 | 0.92 | 669.22314，455.31610，425.30627 | ^[2]^ |
| 15 | Onjisaponin Z | C_71_H_106_O_32_ | 28.39 | 1469.65944 | 1469.66199 | 1.73 | 455.31894，425.30655，  405.14133 | ^[3]^ |
| 16 | Senegasaponin A | C_74_H_110_O_35_ | 28.76 | 1557.67549 | 1557.67419 | 0.83 | 425.30545 | ^[2]^ |
| 17 | Onjisaponin E | C_71_H_106_O_33_ | 28.77 | 1485.65436 | 1485.65552 | 0.78 | 455.31802，425.30652 | ^[2-4]^ |
| 18 | Onjisaponin Ng | C_80_H_118_O_38_ | 28.81 | 1685.72283 | 1685.72485 | 1.20 | 537.18134，455.31985，425.30664 | ^[1, 2, 4]^ |
| 19 | Onjisaponin Qg | C_76_H_120_O_41_ | 28.85 | 1687.72323 | 1687.73157 | 4.95 | 455.31769，425.30670 | ^[3]^ |
| 20 | Onjisaponin 1630 | C_77_H_114_O_37_ | 28.93 | 1629.69662 | 1629.69153 | 3.12 | 669.33875，425.30536，161.04585 | * |
| 21 | Onjisaponin J | C_85_H_126_O_42_ | 29.29 | 1817.76509 | 1817.75806 | 3.87 | 537.18091，455.31628，425.30634 | ^[2, 3]^ |
| 22 | Onjisaponin T | C_83_H_124_O_42_ | 29.50 | 1791.74944 | 1791.74585 | 2.00 | 669.22198，469.15695，237.07736 | ^[3]^ |
| 23 | Onjisaponin F/Wg | C_75_H_112_O_36_ | 30.43 | 1587.68605 | 1587.68250 | 2.24 | 537.18506，455.31650，425.30630 | ^[3]^ |
| 24 | Onjisaponin G | C_70_H_104_O_32_ | 30.62 | 1455.64379 | 1455.64722 | 2.35 | 455.31601，425.30624，237.07698 | ^[3]^ |
| 25 | Onjisaponin Y | C_69_H_102_O_30_ | 32.16 | 1409.63831 | 1409.64478 | 4.59 | 455.31610，425.30649，405.13974 | ^[2, 3]^ |
| 26 | Onjisaponin S | C_81_H_122_O_40_ | 32.65 | 1733.74396 | 1733.73535 | 4.97 | 565.90729，455.31668，425.30594 | ^[3]^ |
| 27 | Onjisaponin W/Fg | C_81_H_120_O_40_ | 32.83 | 1731.72831 | 1731.72742 | 0.51 | 669.22131，583.19110，455.31558，425.30630，237.07655 | ^[3, 8, 10]^ |
| 28 | Onjisaponin Gg | C_76_H_112_O_36_ | 32.91 | 1599.68605 | 1599.68323 | 1.77 | 455.31726，425.30609 | ^[2, 4]^ |
| 29 | Senegasaponin B | C_69_H_102_O_31_ | 33.22 | 1425.63323 | 1425.63794 | 3.31 | 455.31503，425.30634 | ^[2]^ |

* The compound has been identified for the first time.

Tab. S2 Multivariate statistical analysis.

| Group | Ion mode |  | PCA, OPLS-DA | | | Permutation test | |
| --- | --- | --- | --- | --- | --- | --- | --- |
|  |  |  | R^2^X | R^2^Y | Q^2^ | R^2^ | Q^2^ |
| C *vs.* PTS-L | + | PCA | 0.45 | — | 0.235 | — | — |
|  |  | OPLS-DA | 0.62 | 0.987 | 0.763 | (0.0, 0.946) | (0.0, -0.295) |
|  | - | PCA | 0.459 | — | 0.245 | — | — |
|  |  | OPLS-DA | 0.832 | 1 | 0.702 | (0.0, 0.998) | (0.0, -0.265) |
| C *vs.* PTS-H | + | PCA | 0.464 | — | 0.138 | — | — |
|  |  | OPLS-DA | 0.71 | 1 | 0.797 | (0.0, 0.997) | (0.0, 0.0385) |
|  | - | PCA | 0.472 | — | 0.184 | — | — |
|  |  | OPLS-DA | 0.832 | 1 | 0.83 | (0.0, 0.988) | (0.0, -0.0594) |
| C *vs.* DSS | + | PCA | 0.392 | — | 0.172 | — | — |
|  |  | OPLS-DA | 0.537 | 0.991 | 0.875 | (0.0, 0.898) | (0.0, -0.359) |
|  | - | PCA | 0.392 | — | 0.18 | — | — |
|  |  | OPLS-DA | 0.537 | 0.988 | 0.861 | (0.0, 0.902) | (0.0, -0.373) |

Tab. S3 Differential metabolites in mouse feces.

| NO. | *m/z* | Metabolites | Adduct | Formula | MS/MS | C *vs* PTS-L | C *vs* PTS-H | C *vs* DSS |
| --- | --- | --- | --- | --- | --- | --- | --- | --- |
| 1 | 227.0673 | Deoxyuridine | [M-H]^-^ | C_9_H_12_N_2_O_5_ | 66.03456, 94.0297, 124.03999, 182.04617, 184.06133 | ↑* | ↑ | ↑ |
| 2 | 347.2213 | Cortexolone | [M+H]^+^ | C_21_H_30_O_4_ | 107.04906, 119.08549, 123.08065, 137.0956, 147.11678, 159.11665 | ↑** | ↑* | ↑ |
| 3 | 134.0471 | Adenine | [M-H]^-^ | C_5_H_5_N_5_ | 66.00964, 80.02529, 92.0251, 107.03659, 134.04701 | ↓ | ↓ | ↓** |
| 3 | 136.0617 | Adenine | [M+H]^+^ | C_5_H_5_N_5_ | 94.03951, 119.03529, 136.06168 | ↓ | ↓ | ↓** |
| 4 | 514.2833 | Taurocholic acid | [M-H]^-^ | C_26_H_45_NO_7_S | 80.96491, 94.98023, 106.98094, 124.00726, 514.28436 | ↓ | ↓* | ↓** |
| 4 | 533.3237 | Taurocholic acid | [M+NH_4_]^+^ | C_26_H_45_NO_7_S | 67.0545, 69.07001, 93.07043, 95.08563, 108.01146, 126.02132 | ↓ | ↓* | ↓** |
| 5 | 266.088 | Adenosine | [M-H]^-^ | C_10_H_13_N_5_O_4_ | 59.01358, 71.01375, 89.02431, 92.02505, 101.02395, 105.01974 | ↑* | ↑* | ↑ |
| 6 | 363.2163 | Cortisol | [M+H]^+^ | C_21_H_30_O_5_ | 107.0491, 109.0647, 117.07033, 121.06473, 123.08069, 133.06526 | ↑* | ↑** | ↑ |
| 7 | 132.0765 | Creatine | [M+H]^+^ | C_4_H_9_N_3_O_2_ | 72.0445, 74.02382, 90.05515, 114.06568, 132.07639 | ↑ | ↑* | ↑ |
| 8 | 251.0774 | Deoxyinosine | [M-H]^-^ | C_10_H_12_N_4_O_4_ | 59.01382, 73.02923, 83.01355, 85.02925, 89.02386, 99.04514 | ↓ | ↓ | ↓*** |
| 9 | 136.0757 | Dopamine | [M+H-H_2_O]^+^ | C_8_H_11_NO_2_ | 67.05446, 79.05435, 81.07009, 94.06532, 107.04882, 119.04965 | ↓** | ↓* | ↓* |
| 10 | 244.0929 | Cytidine | [M+H]^+^ | C_9_H_13_N_3_O_5_ | 55.01812, 57.03397, 61.02859, 69.04496, 70.02883, 71.01288 | ↓* | ↓* | ↓* |
| 11 | 104.107 | Choline | [M+H]^+^ | C_5_H_14_NO | 58.06521, 60.0811, 104.10662 | ↓ | ↓* | ↓* |
| 12 | 193.035 | D-Glucuronic acid | [M-H]^-^ | C_6_H_10_O_7_ | 59.01377, 71.01393, 73.02913, 75.00874, 87.009, 89.02457 | ↑* | ↑ | ↑ |
| 13 | 165.0401 | Glyceric acid | [M+Hac-H]^-^ | C_3_H_6_O_4_ | 59.01381, 72.99301, 75.00863, 87.00822, 105.01978 | ↓ | ↓* | ↓* |
| 14 | 137.0458 | Hypoxanthine | [M+H]^+^ | C_5_H_4_N_4_O | 110.03473, 119.03511, 137.04541 | ↑ | ↑ | ↑* |
| 15 | 166.086 | Phenylalanine | [M+H]^+^ | C_9_H_11_NO_2_ | 79.05437, 91.05396, 93.06973, 103.05384, 120.08024, 131.04884 | ↓* | ↓ | ↓* |
| 16 | 116.0706 | Proline | [M+H]^+^ | C_5_H_9_NO_2_ | 55.05451, 68.04971, 70.06499, 72.08106, 116.07117 | ↑ | ↓ | ↓* |
| 17 | 120.0653 | L-Threonine | [M+H]^+^ | C_4_H_9_NO_3_ | 56.04961, 58.06525, 74.02385, 74.0601, 84.04422, 85.02833 | ↓ | ↓ | ↓* |
| 18 | 133.0606 | L-Asparagine | [M+H]^+^ | C_4_H_8_N_2_O_3_ | 60.04449, 70.02889, 87.0554, 88.03899, 98.02364, 99.00762 | ↓** | ↓ | ↓ |
| 19 | 179.056 | D-Mannose | [M-H]^-^ | C_6_H_12_O_6_ | 55.01883, 57.03446, 59.01382, 71.01399, 72.99309, 73.02919 | ↓* | ↓* | ↓* |
| 20 | 156.0767 | Histidine | [M+H]^+^ | C_6_H_9_N_3_O_2_ | 68.04972, 81.04453, 83.06048, 93.04483, 95.06041, 110.07087 | ↓ | ↓ | ↓* |
| 21 | 147.1128 | Lysine | [M+H]^+^ | C_6_H_14_N_2_O_2_ | 56.04961, 58.06528, 84.08077, 129.1015, 130.08661, 147.11215 | ↓ | ↓ | ↓** |
| 22 | 327.0924 | Inosine | [M+Hac-H]^-^ | C_10_H_12_N_4_O_5_ | 59.01379, 71.01395, 89.02454, 101.02421, 113.02434, 129.01953 | ↓ | ↑ | ↓*** |
| 23 | 283.2632 | Oleic acid | [M+H]^+^ | C_18_H_34_O_2_ | 53.0388, 55.05458, 57.0703, 67.0545, 69.07003, 71.08561 | ↑* | ↑* | ↑** |
| 24 | 145.0139 | Oxoglutaric acid | [M-H]^-^ | C_5_H_6_O_5_ | 55.01878, 57.03444, 71.0134, 99.0087, 101.0243, 145.01389 | ↑* | ↑* | ↑** |
| 25 | 218.1033 | Pantothenic acid | [M-H]^-^ | C_9_H_17_NO_5_ | 59.01386, 71.01343, 71.05033, 72.00898, 74.02466, 88.04032 | ↑ | ↑* | ↓ |
| 26 | 204.0868 | N-Acetylgalactosamine | [M+H-H_2_O]^+^ | C_8_H_15_NO_6_ | 60.04463, 72.04451, 73.02827, 85.02842, 100.03972, 102.05508 | ↑ | ↑** | ↑ |
| 27 | 222.0968 | N-Acetyl-D-glucosamine | [M+H]^+^ | C_8_H_15_NO_6_ | 60.04462, 72.04451, 73.02827, 102.05508, 120.06527, 186.07478 | ↑** | ↑* | ↑* |
| 28 | 170.0811 | Norepinephrine | [M+H]^+^ | C_8_H_11_NO_3_ | 57.03388, 68.04973, 70.06503, 81.03346, 84.04424, 92.04927 | ↓* | ↓** | ↓* |
| 29 | 147.0662 | Mevalonic acid | [M-H]^-^ | C_6_H_12_O_4_ | 71.01395, 101.06088, 129.05568, 147.06575 | ↑ | ↓ | ↓** |
| 30 | 308.0987 | N-Acetylneuraminic acid | [M-H]^-^ | C_11_H_19_NO_9_ | 58.02962, 59.01382, 71.01386, 72.99303, 84.04517, 87.00829 | ↑* | ↑ | ↓ |
| 31 | 236.0769 | Sepiapterin | [M-H]^-^ | C_9_H_11_N_5_O_3_ | 55.01878, 71.01398, 73.02927, 147.03024, 177.04073, 236.07741 | ↓ | ↓** | ↓* |
| 32 | 187.1079 | Pyridoxine | [M+NH_4_]^+^ | C_8_H_11_NO_3_ | 56.05, 57.03388, 59.04956, 81.03346, 83.04899, 84.04415 | ↓* | ↓* | ↓*** |
| 33 | 377.1458 | Riboflavin | [M+H]^+^ | C_17_H_20_N_4_O_6_ | 61.02885, 73.02861, 117.05469, 172.08679, 198.06758, 200.08153 | ↓ | ↓ | ↓*** |
| 34 | 225.088 | Porphobilinogen | [M-H]^-^ | C_10_H_14_N_2_O_4_ | 59.01374, 181.09903, 225.08929 | ↓* | ↓* | ↓** |
| 34 | 227.1029 | Porphobilinogen | [M+H]^+^ | C_10_H_14_N_2_O_4_ | 136.03908, 138.05522, 139.03897, 181.09738, 209.09074, 227.10446 | ↓* | ↓* | ↓** |
| 35 | 124.0073 | Taurine | [M-H]^-^ | C_2_H_7_NO_3_S | 80.96494, 106.98101, 124.0073 | ↑ | ↓ | ↑* |
| 35 | 126.0221 | Taurine | [M+H]^+^ | C_2_H_7_NO_3_S | 108.01148, 108.9958, 126.02177 | ↑ | ↑ | ↑* |
| 36 | 127.0501 | Thymine | [M+H]^+^ | C_5_H_6_N_2_O_2_ | 54.03407, 55.01812, 56.0497, 57.0339, 82.0285, 84.04423 | ↑** | ↑* | ↑* |
| 37 | 302.3048 | Sphinganine | [M+H]^+^ | C_18_H_39_NO_2_ | 55.05452, 57.07036, 60.04463, 62.06027, 69.06999, 71.08565 | ↑*** | ↑* | ↑ |
| 38 | 149.045 | D-Ribose | [M-H]^-^ | C_5_H_10_O_5_ | 57.03437, 59.01382, 71.01346, 72.99295, 73.02914, 75.00866 | ↓* | ↓* | ↓** |
| 39 | 169.0358 | Uric acid | [M+H]^+^ | C_5_H_4_N_4_O_3_ | 55.02937, 69.00852, 98.03471, 99.01884, 124.01468, 126.02982 | ↑* | ↑* | ↑ |
| 40 | 199.0603 | Vanillylmandelic acid | [M+H]^+^ | C_9_H_10_O_5_ | 53.03894, 55.01813, 81.03347, 93.03383, 95.04911, 97.02799 | ↓ | ↓ | ↓* |
| 41 | 151.0257 | Xanthine | [M-H]^-^ | C_5_H_4_N_4_O_2_ | 65.99844, 108.02001, 133.01537, 151.02565 | ↑** | ↑** | ↑ |
| 42 | 283.0681 | Xanthosine | [M-H]^-^ | C_10_H_12_N_4_O_6_ | 108.02016, 122.0353, 151.02565, 283.0675 | ↑** | ↑ | ↑ |
| 42 | 285.0829 | Xanthosine | [M+H]^+^ | C_10_H_12_N_4_O_6_ | 55.01826, 57.03358, 61.02862, 71.013, 73.02846, 103.03901 | ↑** | ↑ | ↑ |
| 43 | 113.0346 | Uracil | [M+H]^+^ | C_4_H_4_N_2_O_2_ | 70.02884, 96.00805, 113.03425 | ↑* | ↑** | ↑* |
| 44 | 331.2258 | 17-Hydroxyprogesterone | [M+H]^+^ | C_21_H_30_O_3_ | 79.05431, 91.05392, 109.06467, 119.08542, 123.08064, 133.06511 | ↑** | ↑* | ↑ |
| 45 | 407.2791 | 7-Ketodeoxycholic acid | [M+H]^+^ | C_24_H_38_O_5_ | 55.05453, 69.06999, 83.08556, 109.06468, 109.10139, 111.07993 | ↑* | ↑* | ↑ |
| 46 | 423.2758 | 3a,6b,7a,12a-Tetrahydroxy-5b-cholanoic acid | [M-H]^-^ | C_24_H_40_O_6_ | 83.0503, 379.28464, 405.26349, 423.27444 | ↑** | ↑** | ↑ |
| 47 | 424.3044 | 3,7-Dihydroxy-12-oxocholanoic acid | [M+NH_4_]^+^ | C_24_H_38_O_5_ | 55.05454, 69.07002, 83.08565, 93.0701, 95.08569, 109.10119 | ↑ | ↑* | ↑ |
| 48 | 453.2859 | 3a,6b,7b-Trihydroxy-5b-cholanoic acid | [M+FA-H]^-^ | C_24_H_40_O_5_ | 289.21823, 363.29208, 389.27271, 407.2822 | ↑ | ↓ | ↓* |
| 49 | 181.0506 | 3,4-Dihydroxyhydrocinnamic acid | [M-H]^-^ | C_9_H_10_O_4_ | 59.01374, 107.05032, 109.02997, 117.03442, 119.04968, 121.02882 | ↓ | ↓* | ↓ |
| 50 | 373.2736 | 3a,7b,12a-Trihydroxy-5a-Cholanoic acid | [M+H-2H_2_O]^+^ | C_24_H_40_O_5_ | 55.0546, 57.06995, 67.05407, 69.07005, 81.06955, 83.08572 | ↑* | ↑* | ↑ |
| 51 | 405.2637 | 7a,12a-Dihydroxy-3-oxo-4-cholenoic acid | [M+H]^+^ | C_24_H_36_O_5_ | 55.05444, 67.05437, 69.07001, 109.06467, 119.08539, 123.08066 | ↑* | ↑ | ↑ |
| 52 | 160.0614 | Aminoadipic acid | [M-H]^-^ | C_6_H_11_NO_4_ | 58.02971, 59.01371, 70.02953, 71.04974, 74.02467, 85.02924 | ↓ | ↓* | ↓* |
| 53 | 206.0823 | N-Acetyl-L-phenylalanine | [M-H]^-^ | C_11_H_13_NO_3_ | 58.02976, 70.02944, 72.00899, 147.04581, 162.05594, 164.07211 | ↓ | ↓ | ↓** |
| 54 | 175.1192 | L-Arginine | [M+H]^+^ | C_6_H_14_N_4_O_2_ | 60.05599, 70.06501, 112.08714, 114.10258, 116.07001, 133.09734 | ↓*** | ↓ | ↓** |
| 55 | 149.0597 | Cinnamic acid | [M+H]^+^ | C_9_H_8_O_2_ | 79.05441, 103.05395, 105.06947, 131.04889, 149.05873 | ↓** | ↓ | ↓* |
| 56 | 318.0498 | Glucosamine 6-sulfate | [M+Hac-H]^-^ | C_6_H_13_NO_8_S | 71.0134, 80.965, 87.00829, 96.95944 | ↑* | ↑* | ↑ |
| 57 | 451.3413 | Coprocholic acid | [M+H]^+^ | C_27_H_46_O_5_ | 55.0545, 97.10149, 101.05995, 109.10128, 287.20108, 289.21509 | ↑** | ↑ | ↑ |
| 58 | 391.2841 | Cholic acid | [M+H-H_2_O]^+^ | C_24_H_40_O_5_ | 55.05442, 57.07008, 67.05432, 69.06986, 81.06988, 83.08533 | ↑* | ↑* | ↑ |
| 58 | 815.5657 | Cholic acid | [2M-H]^-^ | C_24_H_40_O_5_ | 371.26166, 389.26553, 407.28265 | ↑ | ↑* | ↑ |
| 59 | 195.0505 | Gluconic acid | [M-H]^-^ | C_6_H_12_O_7_ | 59.01377, 71.01394, 72.99308, 73.02913, 75.00854, 87.00898 | ↓ | ↓* | ↓* |
| 60 | 391.2849 | Deoxycholic acid | [M-H]^-^ | C_24_H_40_O_4_ | 327.27124, 343.26404, 345.28009, 391.28357 | ↑** | ↑ | ↑ |
| 61 | 450.322 | Chenodeoxycholic acid glycine conjugate | [M+H]^+^ | C_26_H_43_NO_5_ | 76.03943, 93.06974, 95.08524, 111.07998, 135.11671, 158.08043 | ↑* | ↑* | ↑ |
| 62 | 147.0765 | Glutamine | [M+H]^+^ | C_5_H_10_N_2_O_3_ | 56.04996, 84.04416, 85.02835, 88.03963, 101.07069, 102.05498 | ↓** | ↓ | ↓* |
| 63 | 204.0661 | Indolelactic acid | [M-H]^-^ | C_11_H_11_NO_3_ | 72.9931, 75.00857, 116.05014, 128.05083, 130.06548, 140.05081 | ↓* | ↓** | ↓ |
| 63 | 206.0811 | Indolelactic acid | [M+H]^+^ | C_11_H_11_NO_3_ | 105.06947, 118.06533, 130.06548, 132.08092, 142.06514, 144.08121 | ↓* | ↓** | ↓ |
| 64 | 281.2473 | Linoleic acid | [M+H]^+^ | C_18_H_32_O_2_ | 55.05458, 57.0703, 69.07003, 71.08563, 81.07011, 83.08562 | ↑ | ↑ | ↑* |
| 65 | 150.0584 | Methionine | [M+H]^+^ | C_5_H_11_NO_2_S | 56.04959, 61.01094, 74.02382, 75.02641, 84.04417, 85.02826 | ↓* | ↓* | ↓* |
| 66 | 130.0506 | 4-Hydroxyproline | [M-H]^-^ | C_5_H_9_NO_3_ | 57.03434, 85.0292, 88.03996, 130.05127 | ↓* | ↓** | ↓* |
| 67 | 181.0506 | Hydroxyphenyllactic acid | [M-H]^-^ | C_9_H_10_O_4_ | 72.9931, 93.03458, 107.05016, 117.03444, 119.0497, 135.04488 | ↓ | ↓* | ↑ |
| 68 | 711.2188 | Glycogen | [M+FA-H]^-^ | C_24_H_42_O_21_ | 59.01382, 73.02911, 75.00857, 87.00833, 89.02466, 131.03441 | ↓* | ↓* | ↓** |
| 69 | 187.1088 | Glycylleucine | [M-H]^-^ | C_8_H_16_N_2_O_3_ | 73.04043, 74.02463, 112.0766, 114.09278, 130.08653, 141.10352 | ↓*** | ↓** | ↓*** |
| 69 | 189.1237 | Glycylleucine | [M+H]^+^ | C_8_H_16_N_2_O_3_ | 84.08078, 86.09652, 132.10243, 143.11824, 171.11243, 189.12334 | ↓*** | ↓** | ↓*** |
| 70 | 181.0714 | Mannitol | [M-H]^-^ | C_6_H_14_O_6_ | 59.01382, 71.01345, 73.02918, 87.00827, 89.02386, 101.02437 | ↓ | ↓ | ↓* |
| 71 | 310.1137 | N-Acetyl-a-neuraminic acid | [M+H]^+^ | C_11_H_19_NO_9_ | 60.04455, 61.02861, 73.02827, 85.02851, 116.07001, 130.04996 | ↑* | ↑* | ↓ |
| 72 | 153.0406 | Oxypurinol | [M+H]^+^ | C_5_H_4_N_4_O_2_ | 82.03972, 110.03469, 136.015, 153.04092 | ↑* | ↑* | ↑ |
| 73 | 112.087 | Histamine | [M+H]^+^ | C_5_H_9_N_3_ | 68.04974, 70.06498, 83.06049, 95.06043, 112.08713 | ↓** | ↓*** | ↓*** |
| 74 | 464.2818 | Taurodeoxycholic acid | [M+H-2H_2_O]^+^ | C_26_H_45_NO_6_S | 67.05434, 69.07003, 95.08553, 108.01149, 126.02134, 145.10127 | ↓ | ↓ | ↓** |
| 74 | 498.2887 | Taurodeoxycholic acid | [M-H]^-^ | C_26_H_45_NO_6_S | 80.965, 106.98103, 124.00735, 498.28659 | ↓ | ↓ | ↓** |
| 75 | 391.2842 | Ursocholic acid | [M+H-H_2_O]^+^ | C_24_H_40_O_5_ | 55.05457, 57.07027, 67.05421, 69.07002, 83.04903, 83.08565 | ↑ | ↑ | ↓* |
| 75 | 407.2802 | Ursocholic acid | [M-H]^-^ | C_24_H_40_O_5_ | 57.03436, 289.21832, 343.26508, 371.26157, 389.27271, 407.28253 | ↑ | ↑ | ↓* |
| 76 | 227.1282 | Traumatic acid | [M-H]^-^ | C_12_H_20_O_4_ | 59.01379, 163.11166, 165.12773, 209.11879, 227.12796 | ↓* | ↓* | ↑ |
| 77 | 135.031 | Threonic acid | [M-H]^-^ | C_4_H_8_O_5_ | 71.0134, 87.00752, 89.02383 | ↑ | ↑ | ↑* |
| 78 | 365.2329 | Tetrahydrocortisol | [M-H]^-^ | C_21_H_34_O_5_ | 111.08068, 261.18582, 263.20303, 303.19522, 317.21219, 365.23032 | ↑** | ↑** | ↑ |
| 79 | 464.2839 | Taurochenodesoxycholic acid | [M+H-2H_2_O]^+^ | C_26_H_45_NO_6_S | 93.06956, 108.01149, 126.02174, 208.0648, 464.2851 | ↑ | ↓ | ↓** |
| 79 | 498.2886 | Taurochenodesoxycholic acid | [M-H]^-^ | C_26_H_45_NO_6_S | 80.96498, 94.98112, 106.981, 124.00726, 498.28671 | ↑ | ↓ | ↓** |
| 80 | 188.0551 | Lipoamide | [M+H-H_2_O]^+^ | C_8_H_15_NOS_2_ | 53.03881, 55.05471, 57.07026, 70.02885, 72.0445, 82.06509 | ↓ | ↓ | ↓* |
| 81 | 474.1743 | 10-Formyltetrahydrofolate | [M+H]^+^ | C_20_H_23_N_7_O_7_ | 102.05509, 110.07089, 120.04408, 327.12085 | ↓ | ↓ | ↓* |
| 82 | 178.053 | N-Formyl-L-methionine | [M+H]^+^ | C_6_H_11_NO_3_S | 58.99539, 61.01104, 70.02883, 73.0105, 84.04419, 85.02833 | ↓ | ↓ | ↓** |
| 83 | 381.2282 | Leukotriene B4 | [M+FA-H]^-^ | C_20_H_32_O_4_ | 59.01373, 69.03425, 207.10297, 287.19977, 299.19968, 317.21143 | ↑** | ↑* | ↑ |
| 84 | 182.0811 | 4-Hydroxy-4-(3-pyridyl)-butanoic acid | [M+H]^+^ | C_9_H_11_NO_3_ | 84.08075, 118.06535, 136.07516, 182.08095 | ↓** | ↓* | ↓* |
| 85 | 146.1176 | 4-Trimethylammoniobutanoic acid | [M+H]^+^ | C_7_H_15_NO_2_ | 58.0653, 60.08118, 84.08076, 86.09576, 100.11183, 146.11682 | ↑* | ↑** | ↑** |
| 86 | 160.0755 | Indoleacetaldehyde | [M+H]^+^ | C_10_H_9_NO | 55.05453, 133.06526, 142.06532, 160.07487 | ↑ | ↑ | ↑* |
| 87 | 147.0442 | Coumarin | [M+H]^+^ | C_9_H_6_O_2_ | 97.028, 103.05376, 121.02808, 147.04393 | ↓** | ↓* | ↓* |
| 88 | 351.2174 | Prostaglandin E2 | [M-H]^-^ | C_20_H_32_O_5_ | 287.20056, 289.21823, 305.21347, 307.22543, 333.20718, 351.21646 | ↑* | ↑* | ↑ |
| 89 | 146.165 | Spermidine | [M+H]^+^ | C_7_H_19_N_3_ | 56.04999, 58.06528, 70.06553, 72.08105, 75.09179, 84.08074 | ↑ | ↓ | ↓*** |
| 90 | 503.1605 | Maltotriose | [M-H]^-^ | C_18_H_32_O_16_ | 59.01379, 73.02914, 89.0246, 101.02427, 131.0347, 161.04538 | ↓ | ↓ | ↓** |
| 91 | 188.1757 | N1-Acetylspermidine | [M+H]^+^ | C_9_H_21_N_3_O | 58.0653, 60.08118, 72.04443, 84.08073, 100.07578, 112.11194 | ↑ | ↑ | ↓** |
| 92 | 701.1895 | Maltotetraose | [M+Cl]^-^ | C_24_H_42_O_21_ | 59.01372, 73.02911, 89.02461, 101.02435, 161.0452, 179.05524 | ↓* | ↓** | ↓** |
| 93 | 171.1492 | N6,N6,N6-Trimethyl-L-lysine | [M+H-H_2_O]^+^ | C_9_H_20_N_2_O_2_ | 55.05463, 57.07039, 58.06529, 70.06498, 84.08075, 171.14851 | ↑** | ↑ | ↑ |
| 94 | 279.2316 | alpha-Linolenic acid | [M+H]^+^ | C_18_H_30_O_2_ | 55.05455, 67.05452, 69.07006, 79.05425, 81.07014, 83.08566 | ↑* | ↑* | ↑ |
| 95 | 259.0221 | Glucose 6-phosphate | [M-H]^-^ | C_6_H_13_O_9_P | 59.01373, 71.01395, 96.9694, 101.02427, 138.98026, 168.99117 | ↑ | ↑ | ↑* |
| 96 | 123.0552 | Niacinamide | [M+H]^+^ | C_6_H_6_N_2_O | 53.03889, 55.01814, 79.01765, 123.05479 | ↑ | ↑ | ↓* |
| 97 | 313.1083 | 7,8-Dihydropteroic acid | [M-H]^-^ | C_14_H_14_N_6_O_3_ | 121.02911, 269.11652, 313.11029 | ↑ | ↑* | ↑ |
| 98 | 359.1039 | Pantetheine 4'-phosphate | [M+H]^+^ | C_11_H_23_N_2_O_7_PS | 57.07028, 61.01107, 72.04461, 78.03709, 87.0554, 98.06049 | ↑ | ↑ | ↑* |
| 99 | 300.2894 | 3-Dehydrosphinganine | [M+H]^+^ | C_18_H_37_NO_2_ | 55.05463, 57.06996, 58.02892, 60.04462, 62.06041, 71.08572 | ↑*** | ↑ | ↑** |
| 100 | 124.0393 | Nicotinic acid | [M+H]^+^ | C_6_H_5_NO_2_ | 53.0388, 80.04971, 124.03947 | ↑** | ↑ | ↑ |
| 101 | 384.1506 | N-Acetyllactosamine | [M+H]^+^ | C_14_H_25_NO_11_ | 60.04456, 73.02828, 91.03913, 130.05006, 204.08621, 206.10362 | ↑ | ↑ | ↓*** |
| 102 | 165.0548 | m-Coumaric acid | [M+H]^+^ | C_9_H_8_O_3_ | 53.03881, 79.05437, 91.05399, 95.04905, 103.05392, 119.04858 | ↓** | ↓* | ↓* |
| 103 | 359.2219 | all-trans-Retinoic acid | [M+Hac-H]^-^ | C_20_H_28_O_2_ | 81.03407, 123.04591, 299.19971 | ↑ | ↑** | ↑ |
| 104 | 153.0193 | Protocatechuic acid | [M-H]^-^ | C_7_H_6_O_4_ | 67.01897, 68.99783, 71.01339, 83.01356, 95.0135, 99.00869 | ↓* | ↓*** | ↓ |
| 105 | 183.0297 | 3,4-Dihydroxymandelic acid | [M-H]^-^ | C_8_H_8_O_5_ | 81.03405, 111.04514, 121.02875, 139.03937, 183.03073 | ↓** | ↓** | ↓ |
| 106 | 123.0441 | Benzoic acid | [M+H]^+^ | C_7_H_6_O_2_ | 53.0389, 79.01774, 79.0544, 97.02826, 105.03377, 123.04445 | ↓** | ↓* | ↓* |
| 107 | 293.1144 | Aspartame | [M-H]^-^ | C_14_H_18_N_2_O_5_ | 86.02464, 116.05007, 118.06664, 162.05592, 231.11401, 275.10519 | ↓ | ↓* | ↓ |
| 108 | 591.3192 | Mesobilirubinogen | [M-H]^-^ | C_33_H_44_N_4_O_6_ | 59.01376, 120.04501, 122.06084, 124.07667, 138.09261, 243.14992 | ↑** | ↑* | ↑ |
| 108 | 593.3322 | Mesobilirubinogen | [M+H]^+^ | C_33_H_44_N_4_O_6_ | 285.15775, 468.24893, 593.3316 | ↑** | ↑* | ↑ |
| 109 | 219.1495 | 5-Methoxydimethyltryptamine | [M+H]^+^ | C_13_H_18_N_2_O | 58.06536, 70.06498, 72.08055, 131.04884, 132.08078, 134.05931 | ↑*** | ↑** | ↑ |
| 110 | 145.0616 | Ureidoisobutyric acid | [M-H]^-^ | C_5_H_10_N_2_O_3_ | 102.05574, 127.05072, 145.06192 | ↓** | ↓* | ↓ |
| 111 | 165.0548 | 4-Hydroxycinnamic acid | [M+H]^+^ | C_9_H_8_O_3_ | 65.03881, 91.05403, 95.04911, 103.05395, 119.04863, 121.06471 | ↓** | ↓* | ↓* |
| 112 | 265.2526 | Petroselinic acid | [M+H-H_2_O]^+^ | C_18_H_34_O_2_ | 55.05455, 57.07035, 69.07008, 71.08559, 81.06951, 85.10126 | ↑* | ↑* | ↑** |
| 113 | 282.279 | Palmitoylethanolamide | [M+H-H_2_O]^+^ | C_18_H_37_NO_2_ | 55.05465, 57.07003, 60.04465, 69.07014, 71.08557, 83.0857 | ↑*** | ↑ | ↑* |
| 114 | 282.2791 | Oleamide | [M+H]^+^ | C_18_H_35_NO | 55.05455, 57.07029, 69.07002, 71.0856, 72.04449, 83.08562 | ↑ | ↑ | ↑* |
| 115 | 269.0456 | Apigenin | [M-H]^-^ | C_15_H_10_O_5_ | 63.02366, 107.01353, 117.03456, 151.00278, 269.04501 | ↓* | ↓* | ↓ |
| 116 | 472.1598 | Pteroyl-D-glutamic acid | [M-H]^-^ | C_20_H_23_N_7_O_7_ | 92.05079, 102.05675, 128.03535, 135.05653, 146.04611, 221.09274 | ↓ | ↓ | ↓* |
| 117 | 440.2495 | Leukotriene E4 | [M+H]^+^ | C_23_H_37_NO_5_S | 81.06948, 99.044, 121.10136, 147.11696 | ↓* | ↓* | ↓** |
| 118 | 124.0395 | Picolinic acid | [M+H]^+^ | C_6_H_5_NO_2_ | 53.0388, 80.04971, 97.02888, 124.03947 | ↑** | ↑* | ↑ |
| 119 | 139.0511 | Imidazolepropionic acid | [M-H]^-^ | C_6_H_8_N_2_O_2_ | 66.03457, 93.0456, 94.02962, 95.01359, 95.06104, 121.04034 | ↑ | ↑ | ↓** |
| 120 | 163.061 | 1,5-Anhydrosorbitol | [M-H]^-^ | C_6_H_12_O_5_ | 55.01878, 59.01374, 71.01391, 73.02914, 101.02428, 115.03944 | ↓ | ↓** | ↓** |
| 121 | 349.2022 | 15-Keto-prostaglandin E2 | [M-H]^-^ | C_20_H_30_O_5_ | 57.03441, 59.01328, 97.06563, 111.08067, 179.10748, 205.12166 | ↑** | ↑** | ↑ |
| 121 | 351.2165 | 15-Keto-prostaglandin E2 | [M+H]^+^ | C_20_H_30_O_5_ | 55.05455, 67.05453, 69.07004, 79.0544, 81.06991, 91.05443 | ↑* | ↑** | ↑ |
| 122 | 549.1677 | Raffinose | [M+FA-H]^-^ | C_18_H_32_O_16_ | 59.01381, 71.01343, 73.02919, 75.00857, 87.00831, 89.02384 | ↓* | ↓* | ↓*** |
| 123 | 269.0447 | Genistein | [M-H]^-^ | C_15_H_10_O_5_ | 65.00318, 83.01357, 117.03442, 133.03001, 135.00891, 151.00278 | ↓ | ↓* | ↓** |
| 123 | 271.0599 | Genistein | [M+H]^+^ | C_15_H_10_O_5_ | 68.99712, 111.0078, 119.04901, 121.02807, 153.01779, 253.04863 | ↓ | ↓* | ↓** |
| 124 | 133.0504 | Deoxyribose | [M-H]^-^ | C_5_H_10_O_4_ | 57.03445, 59.01373, 69.03425, 87.00831, 87.04523, 89.02462 | ↓ | ↓ | ↓*** |
| 125 | 255.2314 | Palmitoleic acid | [M+H]^+^ | C_16_H_30_O_2_ | 55.05458, 57.07032, 67.05447, 69.03355, 69.07006, 71.08564 | ↑* | ↑ | ↑ |
| 126 | 247.2418 | Vaccenic acid | [M+H-2H_2_O]^+^ | C_18_H_34_O_2_ | 55.05453, 57.07026, 67.0545, 69.06995, 71.08559, 83.08558 | ↑ | ↑* | ↑* |
| 127 | 273.0767 | Phloretin | [M-H]^-^ | C_15_H_14_O_5_ | 83.01355, 93.03436, 109.02888, 119.04961, 125.02477, 151.00262 | ↑ | ↑ | ↑** |
| 128 | 253.0503 | Daidzein | [M-H]^-^ | C_15_H_10_O_4_ | 65.00309, 91.01871, 117.03448, 133.02875, 135.0089, 211.03906 | ↓* | ↓* | ↓* |
| 129 | 162.0547 | Indole-3-carboxylic acid | [M+H]^+^ | C_9_H_7_NO_2_ | 118.06538, 144.04506, 162.05429 | ↓*** | ↓** | ↓*** |
| 130 | 118.0861 | 5-Aminopentanoic acid | [M+H]^+^ | C_5_H_11_NO_2_ | 53.03881, 55.05459, 56.04968, 70.065, 72.08106, 83.04899 | ↓ | ↓ | ↓* |
| 131 | 175.1077 | N2-Acetylornithine | [M+H]^+^ | C_7_H_14_N_2_O_3_ | 56.0496, 70.06499, 72.04452, 74.02386, 84.0442, 102.05507 | ↓** | ↓** | ↓*** |
| 132 | 159.1131 | (+)-Limonene | [M+Na]^+^ | C_10_H_16_ | 55.05462, 57.07042, 67.0545, 69.07001, 71.08559, 79.05435 | ↓** | ↓*** | ↓*** |
| 133 | 175.1192 | D-Arginine | [M+H]^+^ | C_6_H_14_N_4_O_2_ | 56.0496, 60.05606, 70.06506, 112.08719, 114.05544, 114.10281 | ↓** | ↓** | ↓*** |
| 134 | 146.0922 | 4-Guanidinobutanoic acid | [M+H]^+^ | C_5_H_11_N_3_O_2_ | 55.0182, 56.04959, 57.03387, 58.06529, 60.05597, 61.02859 | ↓ | ↓ | ↑*** |
| 135 | 391.1397 | Gibberellin A3 | [M+FA-H]^-^ | C_19_H_22_O_6_ | 59.01373, 149.02492, 151.04066, 165.05527 | ↓ | ↓ | ↓** |
| 136 | 215.1392 | Dethiobiotin | [M+H]^+^ | C_10_H_18_N_2_O_3_ | 56.04971, 57.07025, 67.05449, 69.07, 81.07014, 83.049 | ↓* | ↓* | ↓ |
| 137 | 115.1116 | 2-Heptanone | [M+H]^+^ | C_7_H_14_O | 53.0388, 55.01812, 55.05453, 57.03384, 57.07027, 59.04956 | ↑* | ↑ | ↑ |
| 138 | 311.2228 | 13-L-Hydroperoxylinoleic acid | [M-H]^-^ | C_18_H_32_O_4_ | 59.01374, 113.09736, 195.14009, 249.22348, 275.20053, 293.20999 | ↓** | ↓* | ↓ |
| 139 | 189.0042 | Oxalosuccinic acid/oxalosuccinate | [M-H]^-^ | C_6_H_6_O_7_ | 59.01374, 71.01396, 72.99304, 99.00828, 127.00436, 189.0041 | ↓ | ↓ | ↑** |
| 140 | 345.2068 | 11-Dehydrocorticosterone | [M+H]^+^ | C_21_H_28_O_4_ | 79.05439, 81.07021, 95.08569, 107.0491, 109.06467, 121.06473 | ↑** | ↑** | ↑ |
| 141 | 227.056 | 3-Methoxy-4-hydroxyphenylglycolaldehyde | [M+FA-H]^-^ | C_9_H_10_O_4_ | 81.03474, 93.03455, 181.05101 | ↓ | ↓** | ↑ |
| 142 | 220.0616 | 4,6-Dihydroxyquinoline | [M+Hac-H]^-^ | C_9_H_7_NO_2_ | 118.0301, 132.04602, 160.04012 | ↓** | ↓*** | ↓** |
| 143 | 222.0407 | 4-(2-Amino-3-hydroxyphenyl)-2,4-dioxobutanoic acid | [M-H]^-^ | C_10_H_9_NO_5_ | 108.04605, 134.02493, 150.05521, 160.04013, 176.03549, 178.0506 | ↓ | ↑ | ↓*** |
| 144 | 188.0705 | 5-Methoxyindoleacetate | [M+H-H_2_O]^+^ | C_11_H_11_NO_3_ | 118.06534, 132.08083, 144.04346, 146.05983, 160.07486, 170.06003 | ↓* | ↓* | ↓* |
| 145 | 591.3153 | Urobilinogen | [M+H]^+^ | C_33_H_42_N_4_O_6_ | 67.05451, 122.06005, 138.09062, 227.11745, 301.15585, 303.16913 | ↑** | ↑ | ↑ |
| 146 | 317.2114 | 12-Keto-leukotriene B4 | [M+H-H_2_O]^+^ | C_20_H_30_O_4_ | 55.05443, 67.05428, 69.03351, 69.06976, 81.06983, 83.08534 | ↑* | ↑ | ↓ |
| 147 | 137.0605 | Tyrosol | [M-H]^-^ | C_8_H_10_O_2_ | 57.03442, 93.03446, 95.05044, 137.06114 | ↓ | ↓** | ↑ |
| 148 | 318.3007 | Phytosphingosine | [M+H]^+^ | C_18_H_39_NO_3_ | 55.05457, 57.07031, 60.04453, 69.07011, 71.08577, 85.1013 | ↑** | ↑* | ↑ |
| 149 | 295.2266 | 13-OxoODE | [M+H]^+^ | C_18_H_30_O_3_ | 53.03881, 55.0546, 57.06995, 67.05406, 69.07008, 71.08564 | ↓** | ↓* | ↓ |
| 150 | 295.2263 | 9-OxoODE | [M+H]^+^ | C_18_H_30_O_3_ | 53.03878, 55.05457, 57.07033, 67.05402, 69.07004, 71.08562 | ↓* | ↓* | ↓ |
| 151 | 381.2266 | 11H-14,15-EETA | [M+FA-H]^-^ | C_20_H_32_O_4_ | 209.11874, 299.19965, 335.22366 | ↑** | ↑** | ↑** |
| 152 | 295.2278 | 9,10-Epoxyoctadecenoic acid | [M-H]^-^ | C_18_H_32_O_3_ | 59.01373, 125.09743, 141.12895, 171.10268, 277.21609, 295.22955 | ↓* | ↓* | ↓ |
| 153 | 329.2336 | 9,12,13-TriHOME | [M-H]^-^ | C_18_H_34_O_5_ | 99.08166, 129.09184, 181.12259, 183.13945, 199.13509, 209.11876 | ↓*** | ↓*** | ↓ |
| 154 | 348.2746 | 9,10,13-TriHOME | [M+NH_4_]^+^ | C_18_H_34_O_5_ | 53.03881, 55.01814, 55.0546, 57.06986, 67.05406, 69.07007 | ↓*** | ↓*** | ↓ |
| 155 | 136.0756 | p-Octopamine | [M+H-H_2_O]^+^ | C_8_H_11_NO_2_ | 67.05452, 94.06537, 107.04916, 109.06487, 118.06542, 119.04864 | ↓** | ↓* | ↓* |
| 156 | 502.2926 | Fexofenadine | [M+H]^+^ | C_32_H_39_NO_4_ | 119.08545, 147.08139, 149.09659, 175.11075, 189.12843 | ↑* | ↑ | ↑* |
| 157 | 281.2471 | (9E,11E)-Octadecadienoic acid | [M+H]^+^ | C_18_H_32_O_2_ | 53.03881, 55.05462, 57.06994, 69.07008, 71.08568, 83.08568 | ↑ | ↑* | ↑ |
| 158 | 281.2473 | (10E,12Z)-Octadecadienoic acid | [M+H]^+^ | C_18_H_32_O_2_ | 55.05456, 57.0703, 67.05452, 69.07004, 71.0857, 81.07013 | ↑ | ↑ | ↑* |
| 159 | 326.2316 | 10-Nitrolinoleic acid | [M+H]^+^ | C_18_H_31_NO_4_ | 55.0545, 57.07022, 67.05447, 69.06998, 71.08559, 81.06943 | ↓* | ↓ | ↓* |
| 160 | 180.1018 | (R)-Salsolinol | [M+H]^+^ | C_10_H_13_NO_2_ | 79.05435, 93.06956, 108.08068, 120.0814, 137.06058, 138.09207 | ↑* | ↑ | ↑ |
| 161 | 255.0664 | Dihydrodaidzein | [M-H]^-^ | C_15_H_12_O_4_ | 65.00311, 93.03458, 119.04957, 121.02893, 133.03017, 135.00894 | ↓ | ↓ | ↓* |
| 162 | 285.0748 | Glycitein | [M+H]^+^ | C_16_H_12_O_5_ | 119.04853, 167.03427, 225.05426, 269.04446, 285.07568 | ↓ | ↓* | ↓** |
| 163 | 301.0718 | Hesperetin | [M-H]^-^ | C_16_H_14_O_6_ | 65.00311, 91.01871, 93.03454, 109.02906, 119.04965, 121.02898 | ↑ | ↑* | ↓ |
| 164 | 165.0911 | Eugenol | [M+H]^+^ | C_10_H_12_O_2_ | 53.03882, 55.01814, 55.05454, 79.05439, 93.06968, 105.03375 | ↓** | ↓ | ↓ |
| 165 | 367.2112 | 20-Carboxy-leukotriene B4 | [M+H]^+^ | C_20_H_30_O_6_ | 55.05453, 95.08517, 137.05908, 137.09561, 149.09662, 177.09004 | ↑* | ↑ | ↑ |
| 165 | 411.202 | 20-Carboxy-leukotriene B4 | [M+FA-H]^-^ | C_20_H_30_O_6_ | 59.01374, 167.10712, 221.11768, 301.18262, 303.19531, 347.18774 | ↑ | ↑** | ↑ |
| 166 | 433.3321 | 24-Hydroxycalcitriol | [M+H]^+^ | C_27_H_44_O_4_ | 55.0546, 71.04924, 105.03374, 123.08067, 203.17993, 269.19113 | ↑* | ↑ | ↑ |
| 167 | 315.1956 | 4-oxo-Retinoic acid | [M+H]^+^ | C_20_H_26_O_3_ | 67.0545, 69.07001, 91.05396, 93.06969, 95.08517, 105.0695 | ↑* | ↑** | ↓ |
| 168 | 425.3422 | alpha-Tocotrienol | [M+H]^+^ | C_29_H_44_O_2_ | 55.05463, 67.05453, 69.07001, 83.08564, 107.08578, 109.10143 | ↑ | ↑ | ↑* |
| 169 | 305.1863 | Dolichol phosphate | [M+H]^+^ | (C_5_H_8_)nC_10_H_21_O_4_P | 55.05456, 83.08562, 97.10145, 121.10133 | ↑** | ↑** | ↑ |
| 170 | 172.0613 | N-Acetyl-L-glutamate 5-semialdehyde | [M-H]^-^ | C_7_H_11_NO_4_ | 58.02974, 67.01844, 72.00895, 74.02464, 84.04513, 88.0403 | ↓* | ↓** | ↓ |
| 170 | 174.076 | N-Acetyl-L-glutamate 5-semialdehyde | [M+H]^+^ | C_7_H_11_NO_4_ | 57.03386, 60.04448, 69.03352, 71.0492, 72.04447, 84.04411 | ↓* | ↓*** | ↓ |
| 171 | 277.2158 | Stearidonic acid | [M+H]^+^ | C_18_H_28_O_2_ | 53.03882, 55.05458, 69.07008, 79.05436, 83.08559, 95.08515 | ↓*** | ↓* | ↓* |
| 172 | 428.14 | Lacto-N-biose I | [M+FA-H]^-^ | C_14_H_29_NO_13_ | 58.02968, 59.01374, 87.00829, 89.02464, 142.05092 | ↑ | ↑ | ↓*** |
| 173 | 319.1901 | Ubiquinone-2 | [M+H]^+^ | C_19_H_26_O_4_ | 69.07002, 83.08564, 93.06964, 107.08569, 121.10136, 135.11641 | ↑* | ↑** | ↑ |
| 173 | 363.1816 | Ubiquinone-2 | [M+FA-H]^-^ | C_19_H_26_O_4_ | 85.02922, 87.04523, 245.15454, 261.14981, 263.16647 | ↑* | ↑ | ↑ |
| 174 | 329.2109 | 21-Hydroxy-5b-pregnane-3,11,20-trione | [M+H-H_2_O]^+^ | C_21_H_30_O_4_ | 55.01813, 79.05442, 81.06959, 91.05398, 95.08523, 107.04907 | ↑** | ↑** | ↑ |
| 175 | 226.0357 | 5-(2'-Carboxyethyl)-4,6-Dihydroxypicolinate | [M-H]^-^ | C_9_H_9_NO_6_ | 59.01372, 69.03478, 72.00895, 83.01353, 109.02886, 113.0244 | ↓* | ↓** | ↓ |
| 175 | 228.0505 | 5-(2'-Carboxyethyl)-4,6-Dihydroxypicolinate | [M+H]^+^ | C_9_H_9_NO_6_ | 87.04383, 111.04442, 112.03903, 122.02293, 124.03932, 136.03918 | ↓* | ↓** | ↓ |
| 176 | 256.0816 | Nicotinic acid ribonucleoside | [M+H]^+^ | C_11_H_14_NO_6_ | 55.01822, 57.03399, 59.04957, 61.02885, 73.02841, 85.02839 | ↓ | ↓** | ↑ |
| 177 | 531.2965 | 5b-Cyprinol sulfate | [M-H]^-^ | C_27_H_48_O_8_S | 80.96492, 96.95954, 531.29657 | ↑** | ↑** | ↑ |
| 177 | 550.3402 | 5b-Cyprinol sulfate | [M+NH_4_]^+^ | C_27_H_48_O_8_S | 71.04865, 91.05392, 127.1116, 417.33557 | ↑*** | ↑ | ↑ |
| 178 | 443.3525 | 4a-Methylzymosterol-4-carboxylic acid | [M+H]^+^ | C_29_H_46_O_3_ | 55.05454, 67.05452, 69.07005, 83.08574, 109.10136, 135.11649 | ↑ | ↑* | ↑* |
| 179 | 295.2264 | 9(S)-HPODE | [M+H-H_2_O]^+^ | C_18_H_32_O_4_ | 53.03882, 55.05459, 57.07044, 67.05406, 69.07005, 71.08565 | ↓*** | ↓*** | ↓ |
| 179 | 313.2371 | 9(S)-HPODE | [M+H]^+^ | C_18_H_32_O_4_ | 53.03882, 55.05458, 57.07027, 67.05406, 69.07005, 71.08564 | ↓*** | ↓*** | ↓ |
| 180 | 621.402 | Cholesterol glucuronide | [M+Hac-H]^-^ | C_33_H_54_O_7_ | 59.01385, 71.01397, 72.99294, 87.00835, 89.02395, 103.00393 | ↑ | ↓ | ↓*** |
| 181 | 522.3549 | LysoPC(18:1(11Z)/0:0) | [M+H]^+^ | C_26_H_52_NO_7_P | 60.08115, 71.08558, 86.09647, 184.0737, 258.10947, 339.29169 | ↓ | ↓* | ↓ |
| 182 | 544.341 | LysoPC(20:4/0:0) | [M+H]^+^ | C_28_H_50_NO_7_P | 60.08075, 86.0965, 184.07376 | ↑* | ↑ | ↑** |
| 183 | 588.3307 | LysoPC(20:4(8Z,11Z,14Z,17Z)/0:0) | [M+FA-H]^-^ | C_28_H_50_NO_7_P | 59.0139, 78.95869, 259.24289, 303.23053 | ↑ | ↑ | ↑** |
| 184 | 506.3611 | LysoPC(P-18:1(9Z)/0:0) | [M+H]^+^ | C_26_H_52_NO_6_P | 57.07032, 58.06528, 60.08115, 71.08558, 85.10124, 86.09647 | ↑* | ↑ | ↓ |
| 185 | 277.1179 | N1-(alpha-D-ribosyl)-5,6-dimethyl-benzimidazole | [M-H]^-^ | C_14_H_18_N_2_O_4_ | 59.01373, 71.01395, 277.11877 | ↓** | ↓ | ↓*** |
| 185 | 279.1342 | N1-(alpha-D-ribosyl)-5,6-dimethyl-benzimidazole | [M+H]^+^ | C_14_H_18_N_2_O_4_ | 120.08019, 262.10794, 279.13446 | ↓** | ↓* | ↓*** |
| 186 | 480.3085 | LysoPE(18:0/0:0) | [M-H]^-^ | C_23_H_48_NO_7_P | 78.95871, 140.01158, 152.99507, 196.03764, 214.04837, 281.24692 | ↓ | ↓ | ↓* |
| 186 | 482.3231 | LysoPE(18:0/0:0) | [M+H]^+^ | C_23_H_48_NO_7_P | 57.03356, 57.06995, 71.08566, 75.04371, 85.10133, 99.11707 | ↓ | ↓ | ↓* |
| 187 | 514.2833 | Taurohyocholate | [M-H]^-^ | C_26_H_45_NO_7_S | 80.965, 94.98025, 106.981, 124.0073, 514.28418 | ↓ | ↓ | ↓** |
| 187 | 516.2985 | Taurohyocholate | [M+H]^+^ | C_26_H_45_NO_7_S | 55.05463, 93.06956, 108.01138, 111.07991, 126.02174, 208.06467 | ↓ | ↓ | ↓** |
| 188 | 271.1405 | 2-(3-Carboxy-3-(methylammonio)propyl)-L-histidine | [M+H]^+^ | C_11_H_19_N_4_O_4_ | 56.04961, 60.04451, 72.04451, 83.06051, 88.03899, 114.05544 | ↓* | ↓* | ↓* |
| 189 | 522.2039 | Melezitose | [M+NH_4_]^+^ | C_18_H_32_O_16_ | 91.03909, 103.03857, 145.04887, 163.06062 | ↓* | ↓** | ↓** |
| 190 | 190.0542 | N-Acetyl-L-methionine | [M-H]^-^ | C_7_H_13_NO_3_S | 98.06065, 100.04069, 142.05083, 148.04317, 190.05336 | ↓ | ↓* | ↓* |
| 190 | 192.069 | N-Acetyl-L-methionine | [M+H]^+^ | C_7_H_13_NO_3_S | 61.01097, 74.02386, 75.02641, 85.02832, 87.02619, 100.07575 | ↓* | ↓* | ↓* |
| 191 | 172.0978 | N-Acetyl-Leu | [M-H]^-^ | C_8_H_15_NO_3_ | 58.02962, 128.10834, 130.08649, 172.09816 | ↓** | ↓** | ↓** |
| 192 | 367.212 | 5(6)-Epoxy Prostaglandin E1 | [M-H]^-^ | C_20_H_32_O_6_ | 83.05014, 97.06635, 181.12259, 367.21094 | ↑* | ↑ | ↑ |
| 193 | 220.1182 | Zeatin | [M+H]^+^ | C_10_H_13_N_5_O | 55.05452, 59.04922, 67.05453, 69.07002, 83.049, 85.06471 | ↑ | ↑* | ↓ |
| 194 | 165.0546 | Enol-phenylpyruvate | [M+H]^+^ | C_9_H_8_O_3_ | 91.05389, 93.06958, 103.05387, 119.04846, 147.04419, 165.0538 | ↓ | ↓** | ↑ |
| 195 | 115.0401 | Glutarate semialdehyde | [M-H]^-^ | C_5_H_8_O_3_ | 59.01374, 87.04526, 115.0406 | ↑* | ↑ | ↑ |
| 196 | 116.0351 | L-Aspartate-semialdehyde | [M-H]^-^ | C_4_H_7_NO_3_ | 72.0455, 74.0247, 98.0248, 116.03458 | ↓ | ↓ | ↓** |
| 197 | 299.2003 | all-trans-18-Hydroxyretinoic acid | [M+H-H_2_O]^+^ | C_20_H_28_O_3_ | 53.03877, 55.05466, 67.05408, 81.06953, 83.08569, 93.06963 | ↑* | ↑ | ↑ |
| 198 | 351.2166 | Prostaglandin H3 | [M+H]^+^ | C_20_H_30_O_5_ | 55.05452, 67.05449, 69.07, 79.05449, 81.06947, 91.05393 | ↑* | ↑ | ↑ |
| 198 | 351.2167 | Prostaglandin H3 | [M+H]^+^ | C_20_H_30_O_5_ | 53.03881, 55.05454, 67.05452, 69.03356, 69.07005, 79.05438 | ↑** | ↑ | ↑ |
| 199 | 365.2331 | Beta-Cortolone | [M-H]^-^ | C_21_H_34_O_5_ | 193.12399, 285.18402, 365.23035 | ↑** | ↑** | ↑ |
| 200 | 154.0975 | N-Acetylhistamine | [M+H]^+^ | C_7_H_11_N_3_O | 56.04964, 68.04974, 74.06012, 83.06036, 95.06043, 110.05991 | ↓* | ↓** | ↓* |
| 201 | 205.0965 | D-Tryptophan | [M+H]^+^ | C_11_H_12_N_2_O_2_ | 74.02373, 118.0653, 130.06566, 132.08076, 142.06531, 144.0811 | ↓* | ↓* | ↓* |
| 202 | 322.2734 | Alpha-Linolenoyl ethanolamide | [M+H]^+^ | C_20_H_35_NO_2_ | 55.05453, 67.05447, 69.06999, 79.05439, 81.07009, 83.08558 | ↑ | ↑* | ↑ |
| 203 | 355.2624 | Cervonoyl ethanolamide | [M+H-H_2_O]^+^ | C_24_H_36_O_3_ | 67.05459, 69.07017, 79.05424, 81.07018, 83.08569, 95.08512 | ↑* | ↑* | ↑ |
| 204 | 153.0189 | 3,5-Dihydroxybenzoic acid | [M-H]^-^ | C_7_H_6_O_4_ | 67.01897, 109.02962, 135.0087, 153.01981 | ↓ | ↓** | ↓ |
| 205 | 213.1229 | Butabarbital | [M+H]^+^ | C_10_H_16_N_2_O_3_ | 55.05454, 57.07027, 67.05452, 69.07002, 93.0696, 109.10144 | ↓*** | ↓** | ↓*** |
| 206 | 103.0542 | Cycloserine | [M+H]^+^ | C_3_H_6_N_2_O_2_ | 55.01814, 60.0446, 72.04451 | ↓** | ↓ | ↓* |
| 207 | 307.2013 | Hyoscyamine | [M+NH_4_]^+^ | C_17_H_23_NO_3_ | 81.07013, 83.0856, 91.05392, 105.06942, 108.08053, 110.09606 | ↑** | ↑** | ↑ |
| 208 | 263.1395 | Methohexital | [M+H]^+^ | C_14_H_18_N_2_O_3_ | 55.05453, 79.05437, 83.08564, 91.05391, 93.06959, 119.08554 | ↓** | ↓* | ↓*** |
| 209 | 237.16 | Procaine | [M+H]^+^ | C_13_H_20_N_2_O_2_ | 67.05449, 70.06498, 72.08104, 82.06507, 84.08064, 86.09653 | ↓* | ↓* | ↑ |
| 210 | 321.0727 | Cinoxacin | [M+Hac-H]^-^ | C_12_H_10_N_2_O_5_ | 144.04494, 160.04018, 187.05017 | ↓ | ↓ | ↓** |
| 211 | 245.0931 | Methylphenobarbital | [M-H]^-^ | C_13_H_14_N_2_O_3_ | 58.02971, 186.05484, 245.09311 | ↓ | ↓** | ↓* |
| 212 | 361.2007 | Prednisolone | [M+H]^+^ | C_21_H_28_O_5_ | 107.04879, 111.07992, 121.06468, 133.06548, 135.08086, 159.08054 | ↑** | ↑ | ↑ |
| 213 | 295.1038 | Didanosine | [M+Hac-H]^-^ | C_10_H_12_N_4_O_3_ | 55.01878, 59.01403, 73.02972, 133.01685, 179.0553, | ↓* | ↓* | ↓* |
| 214 | 206.1417 | Phenformin | [M+H]^+^ | C_10_H_15_N_5_ | 51.02341, 53.03888, 77.03838 | ↑*** | ↑ | ↑ |
| 215 | 385.1427 | Mesoridazine | [M-H]^-^ | C_21_H_26_N_2_OS_2_ | 96.08176, 106.06552, 385.14627 | ↑** | ↑** | ↑ |
| 216 | 241.1545 | Pirbuterol | [M+H]^+^ | C_12_H_20_N_2_O_3_ | 58.06542, 60.0445, 69.03355, 70.06503, 72.08066, 86.09651 | ↓ | ↓* | ↓* |
| 217 | 575.3574 | Fusidic Acid | [M+Hac-H]^-^ | C_31_H_48_O_6_ | 59.01373, 83.01354, 85.02925 | ↑ | ↓ | ↓* |
| 218 | 196.0965 | Tyrosine methylester | [M+H]^+^ | C_10_H_13_NO_3_ | 79.05437, 81.03348, 81.06947, 91.05396, 94.06535, 95.04904 | ↑* | ↑ | ↓ |
| 219 | 331.0812 | 3,7-Dimethylquercetin | [M+H]^+^ | C_17_H_14_O_7_ | 153.01767, 273.03964, 285.03909, 287.05316, 299.05521, 303.04822 | ↓ | ↑ | ↓* |
| 220 | 162.0763 | Acetylhomoserine | [M+H]^+^ | C_6_H_11_NO_4_ | 56.04961, 57.03387, 58.06571, 61.02855, 69.03354, 71.04922 | ↑** | ↑ | ↓ |
| 221 | 315.1198 | Hypoglycin B | [M+FA-H]^-^ | C_12_H_18_N_2_O_5_ | 86.02464, 102.05537, 128.03534, 157.06209 | ↓* | ↓* | ↓* |
| 222 | 245.1133 | Nopalinic acid | [M+H-H_2_O]^+^ | C_10_H_18_N_2_O_6_ | 55.01818, 56.04957, 58.0653, 70.06501, 71.01289, 73.02824 | ↓*** | ↓* | ↓*** |
| 223 | 401.1243 | Nobiletin | [M-H]^-^ | C_21_H_22_O_8_ | 55.01878, 57.03436, 85.0292 | ↓* | ↓* | ↓** |
| 224 | 347.2213 | (+)-Calycanthine | [M+H]^+^ | C_22_H_26_N_4_ | 81.07014, 83.08562, 347.22061 | ↑** | ↑* | ↑ |
| 225 | 209.1172 | gamma-Asarone | [M+H]^+^ | C_12_H_16_O_3_ | 53.0388, 57.03358, 59.04913, 95.04921, 107.04907, 109.06463 | ↓* | ↓* | ↓** |
| 226 | 174.1489 | (2S,4R,5S)-Muscarine | [M+H]^+^ | C_9_H_20_NO_2_ | 55.05465, 57.03348, 57.06985, 58.06532, 60.08117, 71.04923 | ↓ | ↓*** | ↓*** |
| 227 | 549.1679 | Panose | [M+FA-H]^-^ | C_18_H_32_O_16_ | 59.01381, 71.01344, 73.02914, 75.00857, 85.02925, 87.00837 | ↓** | ↓** | ↓*** |
| 228 | 251.0765 | Nebularine | [M-H]^-^ | C_10_H_12_N_4_O_4_ | 59.01373, 71.01373, 87.00877, 89.02433, 101.02406, 113.02441 | ↓ | ↓ | ↓** |
| 229 | 439.3207 | Neotigogenin | [M+Na]^+^ | C_27_H_44_O_3_ | 55.05457, 57.07025, 67.05453, 69.07004, 71.08559, 125.097 | ↑ | ↑* | ↑ |
| 230 | 162.0916 | (R)-Boschniakine | [M+H]^+^ | C_10_H_11_NO | 55.05454, 93.07045, 105.06949, 106.06532, 117.07033, 130.06554 | ↑*** | ↑ | ↓ |
| 231 | 285.1129 | Sativan | [M-H]^-^ | C_17_H_18_O_4_ | 107.05025, 121.02895, 123.04512, 147.04539, 149.0612, 225.09322 | ↑* | ↑** | ↑ |
| 232 | 389.1245 | trans-Piceid | [M-H]^-^ | C_20_H_22_O_8_ | 59.01378, 73.02912, 93.03455, 109.02888, 271.09875 | ↓ | ↑ | ↓** |
| 233 | 163.0397 | cis-p-Coumaric acid | [M-H]^-^ | C_9_H_8_O_3_ | 93.03485, 117.03442, 119.04964, 121.02898, 163.0406 | ↓ | ↓ | ↓* |
| 234 | 255.0644 | 5,7-Dihydroxyisoflavone | [M+H]^+^ | C_15_H_10_O_4_ | 119.04853, 121.02802, 137.02261, 237.05423, 255.06494 | ↓* | ↓* | ↓* |
| 235 | 327.0869 | Betagarin | [M-H]^-^ | C_18_H_16_O_6_ | 93.03423, 107.05026, 131.05002, 133.0663, 283.09799, 309.07739 | ↑* | ↑** | ↑ |
| 236 | 275.0915 | (-)-Epiafzelechin | [M+H]^+^ | C_15_H_14_O_5_ | 53.0388, 55.01813, 79.05437, 95.04903, 107.04907, 119.04855 | ↑ | ↑ | ↑** |
| 237 | 273.0766 | Afzelechin | [M-H]^-^ | C_15_H_14_O_5_ | 57.03447, 63.02364, 83.01366, 93.03457, 109.02887, 123.04513 | ↓ | ↓** | ↓* |
| 237 | 275.0914 | Afzelechin | [M+H]^+^ | C_15_H_14_O_5_ | 53.0388, 55.01812, 69.03354, 95.04902, 107.04901, 119.04977 | ↓ | ↓* | ↓* |
| 238 | 563.1408 | Apiin | [M-H]^-^ | C_26_H_28_O_14_ | 71.0134, 93.03458, 117.03442, 295.06311, 311.05551, 323.05646 | ↓ | ↓* | ↓** |
| 239 | 293.211 | (2'E,4'Z,7'Z,8E)-Colnelenic acid | [M+H]^+^ | C_18_H_28_O_3_ | 55.05463, 69.07011, 79.05452, 83.0856, 93.06969, 95.08515 | ↓** | ↓** | ↓ |
| 240 | 269.2471 | Cyclohexaneundecanoic acid | [M+H]^+^ | C_17_H_32_O_2_ | 53.03881, 55.05461, 57.07034, 67.05458, 69.07008, 71.08567 | ↑ | ↑ | ↑* |
| 241 | 269.2469 | 9E-Heptadecenoic acid | [M+H]^+^ | C_17_H_32_O_2_ | 53.03916, 55.05439, 57.07012, 67.05431, 69.06982, 71.08538 | ↑ | ↑* | ↑ |
| 242 | 176.128 | Ethyl 3-oxohexanoate | [M+NH_4_]^+^ | C_8_H_14_O_3_ | 55.05461, 61.02859, 69.03345, 71.04928, 73.02824, 85.02835 | ↓ | ↓ | ↓** |
| 243 | 210.1849 | trans-Isoasarone | [M+H]^+^ | C_12_H_16_O_3_ | 95.04904, 109.06469, 123.08067 | ↑ | ↑ | ↑** |
| 244 | 194.1171 | 2-Isopropylphenyl methylcarbamate | [M+H]^+^ | C_11_H_15_NO_2_ | 57.07027, 67.05451, 69.07002, 79.05437, 91.05396, 93.0696 | ↓* | ↓* | ↓* |
| 245 | 102.1277 | Hexylamine | [M+H]^+^ | C_6_H_15_N | 55.05449, 57.07028, 58.06532, 60.08122, 72.0807, 74.09664 | ↑ | ↑ | ↑*** |
| 246 | 211.1331 | Jasmonic acid | [M+H]^+^ | C_12_H_18_O_3_ | 55.05449, 57.07029, 67.05447, 69.06998, 79.05428, 81.07014 | ↓* | ↓* | ↑ |
| 247 | 176.0385 | (R)C(S)S-Alliin | [M-H]^-^ | C_6_H_11_NO_3_S | 60.97538, 70.02995, 176.03789 | ↓ | ↓ | ↓** |
| 248 | 591.1764 | Pelargonidin 3-sophoroside | [M-H]^-^ | C_28_H_33_O_14_ | 55.01878, 57.03437, 59.0138, 71.01398, 73.02914, 85.02924 | ↓ | ↓ | ↓*** |
| 249 | 449.107 | Astilbin | [M-H]^-^ | C_21_H_22_O_11_ | 59.01375, 71.01344, 73.02914, 87.0083, 89.02386 | ↑ | ↓ | ↓** |
| 250 | 306.279 | Octadecanamide | [M+Na]^+^ | C_18_H_37_NO | 55.05468, 57.06987, 69.07013, 71.08562, 83.08582, 86.06017 | ↓*** | ↓*** | ↓* |
| 251 | 118.0651 | Benzeneacetonitrile | [M+H]^+^ | C_8_H_7_N | 53.03898, 91.05431, 118.06538 | ↑** | ↑ | ↓ |
| 252 | 333.1335 | Byssochlamic acid | [M+H]^+^ | C_18_H_20_O_6_ | 55.05453, 67.05446, 69.07001, 177.05429, 227.07201, 285.11215 | ↑ | ↑** | ↑ |
| 253 | 299.258 | Ricinoleic acid | [M+H]^+^ | C_18_H_34_O_3_ | 55.05453, 57.07027, 67.0545, 69.07003, 71.08556, 81.07008 | ↑** | ↑ | ↑* |
| 254 | 153.0193 | Patulin | [M-H]^-^ | C_7_H_6_O_4_ | 91.01871, 109.0289, 153.02007 | ↓ | ↓* | ↓ |
| 255 | 173.0928 | L-Theanine | [M-H]^-^ | C_7_H_14_N_2_O_3_ | 74.02466, 127.08744, 129.10265, 173.09369 | ↓** | ↓** | ↓*** |
| 256 | 211.0612 | Valtrate | [M-H]^-^ | C_10_H_12_O_5_ | 121.02905, 137.02463, 165.05499 | ↓ | ↓ | ↓* |
| 257 | 423.3612 | Soyasapogenol C | [M+H-H_2_O]^+^ | C_30_H_48_O_2_ | 57.07027, 67.05452, 69.06982, 83.08566, 121.10088, 123.11657 | ↓ | ↓* | ↓** |
| 258 | 943.525 | Soyasaponin I | [M+H]^+^ | C_48_H_78_O_18_ | 75.04433, 87.04379, 129.05405, 147.06436, 163.06076, 165.12775 | ↓* | ↓* | ↓** |
| 258 | 987.514 | Soyasaponin I | [M+FA-H]^-^ | C_48_H_78_O_18_ | 71.01342, 73.02914, 87.00831, 87.0452, 89.02464, 103.03956 | ↓* | ↓** | ↓** |
| 259 | 499.3057 | Cucurbitacin D | [M+H-H_2_O]^+^ | C_30_H_44_O_7_ | 67.05451, 69.06998, 137.0592, 139.07471, 283.17242, 357.20657 | ↑** | ↑* | ↑ |
| 260 | 722.3954 | Fumonisin B1 | [M+H]^+^ | C_34_H_59_NO_15_ | 57.06996, 85.02834, 85.10127, 97.10146, 113.02382, 159.02913 | ↑ | ↑ | ↑** |
| 261 | 294.0984 | Sambunigrin | [M-H]^-^ | C_14_H_17_NO_6_ | 85.02918, 87.00907, 101.02432, 294.09781 | ↓ | ↓ | ↓* |
| 262 | 363.1815 | Cinncassiol C2 | [M-H]^-^ | C_20_H_28_O_6_ | 57.03436, 83.05013, 95.05024, 363.18039 | ↑** | ↑** | ↑ |
| 263 | 165.0909 | 4-Isopropylbenzoic acid | [M+H]^+^ | C_10_H_12_O_2_ | 67.05416, 79.05436, 91.05477, 119.08544, 165.0919 | ↑ | ↓ | ↓*** |
| 264 | 293.1761 | Phytuberin | [M-H]^-^ | C_17_H_26_O_4_ | 233.15331, 275.16589, 293.17673 | ↓ | ↓ | ↓* |
| 265 | 227.0915 | Genipin | [M+H]^+^ | C_11_H_14_O_5_ | 53.0388, 55.01812, 95.04903, 107.04914, 123.04413, 125.05987 | ↑ | ↓ | ↓* |
| 266 | 265.1439 | Vulgarin | [M+H]^+^ | C_15_H_20_O_4_ | 53.03881, 55.0546, 69.03354, 71.04925, 93.06956, 97.028 | ↓ | ↓ | ↓* |
| 267 | 679.2991 | 4-Deacetylneosolaniol | [2M-H]^-^ | C_17_H_24_O_7_ | 95.01357, 99.00867, 113.02466 | ↓ | ↓ | ↓** |
| 268 | 285.0759 | (-)-Maackiain | [M+H]^+^ | C_16_H_12_O_5_ | 139.03896, 163.03899, 225.05424, 285.07571 | ↓* | ↓ | ↓ |
| 269 | 249.1488 | Artabsin | [M+H]^+^ | C_15_H_20_O_3_ | 53.03881, 55.05453, 57.07026, 81.07015, 83.08563, 105.06947 | ↑** | ↑* | ↓ |
| 270 | 957.5045 | Asiaticoside | [M-H]^-^ | C_48_H_78_O_19_ | 73.02914, 87.00826, 87.04528, 89.0246, 103.03954, 145.05008 | ↓* | ↓* | ↓** |
| 270 | 959.5231 | Asiaticoside | [M+H]^+^ | C_48_H_78_O_19_ | 83.08561, 87.0438, 129.05414, 163.06059 | ↓* | ↓* | ↓** |
| 271 | 333.2056 | ent-7alpha,12beta-Dihydroxy-16-kauren-19,6beta-olide | [M+H]^+^ | C_20_H_28_O_4_ | 79.05447, 95.04905, 105.06948, 117.07019, 119.08539, 133.06528 | ↑* | ↑ | ↑ |
| 272 | 213.1486 | Traumatin | [M+H]^+^ | C_12_H_20_O_3_ | 53.03881, 55.01814, 55.05461, 67.05455, 69.07005, 73.02827 | ↓*** | ↓*** | ↓ |
| 273 | 591.176 | Isowertin 2''-rhamnoside | [M-H]^-^ | C_28_H_32_O_14_ | 55.01877, 57.03448, 59.01379, 71.0134, 73.02917, 87.00834 | ↓ | ↓ | ↓*** |
| 274 | 137.1322 | beta-Myrcene | [M+H]^+^ | C_10_H_16_ | 53.0388, 55.05457, 57.06991, 65.03879, 69.07008, 79.05438 | ↑ | ↑ | ↑* |
| 275 | 226.1074 | 6-Benzylaminopurine | [M+H]^+^ | C_12_H_11_N_5_ | 79.05437, 91.05395, 93.06958, 226.10793 | ↑* | ↑ | ↓ |
| 276 | 941.5075 | Dehydrosoyasaponin I | [M+H]^+^ | C_48_H_76_O_18_ | 87.0446, 129.05412, 147.06441, 163.06064, 163.11218, 165.12811 | ↓ | ↓ | ↓** |
| 277 | 941.508 | Arvensoside D | [M-H]^-^ | C_48_H_78_O_18_ | 73.02912, 89.0246, 101.02422, 103.0396, 161.0452, 437.34143 | ↓* | ↓** | ↓** |
| 278 | 315.1241 | Sorgolactone | [M-H]^-^ | C_18_H_20_O_5_ | 81.03474, 87.04523, 297.11368, 315.12241 | ↑ | ↑* | ↑ |
| 278 | 334.1651 | Sorgolactone | [M+NH_4_]^+^ | C_18_H_20_O_5_ | 55.01812, 67.0545, 69.07001, 71.04922, 79.05436, 93.06957 | ↑* | ↑* | ↑ |
| 279 | 251.1646 | Abscisic alcohol | [M+H]^+^ | C_15_H_22_O_3_ | 55.05437, 57.07011, 67.05432, 69.06982, 71.04922, 71.08615 | ↓ | ↓ | ↓** |
| 280 | 315.2528 | Dibutyl decanedioate | [M+H]^+^ | C_18_H_34_O_4_ | 55.05461, 57.06993, 69.03355, 69.07005, 83.08564, 97.10136 | ↓* | ↓ | ↓* |
| 281 | 409.1771 | Quinaprilat | [M-H]^-^ | C_23_H_26_N_2_O_5_ | 161.06068, 247.11003, 409.17563 | ↓** | ↓ | ↑* |
| 282 | 180.1019 | Salsolinol | [M+H]^+^ | C_10_H_13_NO_2_ | 106.06532, 108.08072, 120.08035, 138.09216, 162.09149, 180.10068 | ↑* | ↑ | ↑ |
| 283 | 121.0657 | 3-Ethylphenol | [M-H]^-^ | C_8_H_10_O | 65.03941, 93.03458, 121.06561 | ↓ | ↓ | ↓* |
| 284 | 495.118 | 1-Nitro-5-glutathionyl-6-hydroxy-5,6-dihydronaphthalene | [M-H]^-^ | C_20_H_24_N_4_O_9_S | 57.03434, 59.01375, 72.00893, 74.02467, 84.04518, 102.05562 | ↑ | ↓ | ↓* |
| 285 | 213.1228 | 2-Phenyl-1,3-propanediol monocarbamate | [M+NH_4_]^+^ | C_10_H_13_NO_3_ | 55.01814, 57.03389, 76.03942, 81.07017, 83.08565, 166.08629 | ↓*** | ↓** | ↓*** |
| 286 | 313.2385 | 9,10-Epoxyoctadecanoic acid | [M-H]^-^ | C_18_H_34_O_4_ | 59.01373, 97.06544, 113.09624, 195.13754, 277.21646, 295.22501 | ↑ | ↑** | ↓ |
| 287 | 134.06 | 1,3-Dihydro-(2H)-indol-2-one | [M+H]^+^ | C_8_H_7_NO | 79.05379, 91.05397, 106.0653, 134.05934 | ↑ | ↑* | ↑* |
| 288 | 311.2229 | 11-HpODE | [M-H]^-^ | C_18_H_32_O_4_ | 59.01374, 125.09716, 139.11208, 151.11194, 155.10745, 167.10918 | ↓** | ↓** | ↓ |
| 289 | 377.1963 | 5,6-epoxy,18R-HEPE | [M+FA-H]^-^ | C_20_H_28_O_4_ | 57.03436, 69.03424, 83.05013, 243.13937, 257.15405, 287.16281 | ↑* | ↑ | ↑ |
| 290 | 330.2631 | (10E,12Z)-(9S)-9-Hydroperoxyoctadeca-10,12-dienoic acid | [M+NH_4_]^+^ | C_18_H_32_O_4_ | 55.05462, 57.07027, 67.05406, 69.07014, 71.08567, 81.06958 | ↓*** | ↓* | ↓ |
| 291 | 299.091 | Apigenin 7,4'-dimethyl ether | [M+H]^+^ | C_17_H_14_O_5_ | 103.05488, 107.0491, 133.06529, 137.02258, 187.03899, 193.04904 | ↑ | ↑ | ↑** |
| 292 | 144.0805 | 1-Naphthylamine | [M+H]^+^ | C_10_H_9_N | 67.0545, 79.05437, 118.06535 | ↓* | ↓* | ↓* |
| 293 | 159.0913 | 1,5-Naphthalenediamine | [M+H]^+^ | C_10_H_10_N_2_ | 57.06987, 91.05397, 115.05437, 118.06643, 130.06554, 132.0809 | ↓* | ↓* | ↓* |
| 294 | 297.2431 | 9,10-Epoxystearic acid | [M-H]^-^ | C_18_H_34_O_3_ | 57.03437, 139.11246, 155.14227, 253.25304, 297.2413 | ↑** | ↑ | ↑ |
| 295 | 256.0592 | Thiobencarb | [M-H]^-^ | C_12_H_16_ClNOS | 82.02964, 126.00334, 256.05862 | ↑** | ↑** | ↑** |
| 296 | 182.081 | D-Tyrosine | [M+H]^+^ | C_9_H_11_NO_3_ | 95.04912, 107.05015, 109.0647, 118.06538, 119.04855, 121.06481 | ↓** | ↓* | ↓* |
| 297 | 301.0718 | Homoeriodictyol | [M-H]^-^ | C_16_H_14_O_6_ | 93.03452, 107.05022, 123.04515, 259.06119, 271.06094, 301.06857 | ↑** | ↑* | ↑ |
| 298 | 302.1357 | N-(N-(3-Amino-3-carboxypropyl)-3-amino-3-carboxypropyl)azetidine-2-carboxylic acid | [M-H]^-^ | C_12_H_21_N_3_O_6_ | 71.01395, 72.00897, 85.02924, 88.04031, 99.05629, 100.0406 | ↓ | ↓ | ↓** |
| 299 | 159.1493 | N-Nitrosodibutylamine | [M+H]^+^ | C_8_H_18_N_2_O | 55.05452, 57.07028, 72.08104, 84.08074, 86.09649, 159.14911 | ↑* | ↑** | ↑ |
| 300 | 264.0724 | O-Succinyl-L-homoserine | [M+FA-H]^-^ | C_8_H_13_NO_6_ | 72.00894, 112.04059, 124.03997 | ↓** | ↓** | ↓* |
| 301 | 309.2071 | 13(S)-Hydroperoxylinolenic acid | [M-H]^-^ | C_18_H_30_O_4_ | 59.01372, 97.06543, 111.08068, 153.09189, 155.10747, 193.12363 | ↓** | ↓** | ↓ |
| 302 | 313.2385 | 9,10-Epoxy-18-hydroxy-octadecanoic acid | [M-H]^-^ | C_18_H_34_O_4_ | 59.01374, 125.09615, 127.1119, 139.11246, 155.10779, 157.1237 | ↓* | ↓ | ↓* |
| 303 | 315.2537 | 9,10-Dihydroxystearic acid | [M-H]^-^ | C_18_H_36_O_4_ | 127.11321, 141.12895, 297.24112, 315.25424 | ↑ | ↑* | ↑** |
| 304 | 293.088 | Tuliposide B | [M-H]^-^ | C_11_H_18_O_9_ | 59.01378, 69.03426, 71.01395, 85.02925, 87.00896, 89.02463 | ↓*** | ↓* | ↓*** |
| 305 | 363.1452 | Gibberellin A8 | [M-H]^-^ | C_19_H_24_O_7_ | 59.01374, 303.12521, 363.14774 | ↑ | ↑* | ↑ |
| 306 | 375.1444 | Gibberellin A5 | [M+FA-H]^-^ | C_19_H_22_O_5_ | 135.04494, 147.04546, 193.08566 | ↑ | ↓ | ↓** |
| 307 | 241.1078 | Dihydroconiferyl alcohol | [M+Hac-H]^-^ | C_10_H_14_O_3_ | 121.06559, 135.08182, 163.0761, 181.08595 | ↓** | ↓** | ↑ |
| 308 | 218.982 | 1-deoxy-L-glycero-tetrulose 4-phosphate | [M+Cl]^-^ | C_4_H_9_O_6_P | 59.01372, 71.01337, 85.02921 | ↓ | ↓ | ↓* |

Tab. S4 The reliability level to investigate the mechanism of intestinal toxicity upon toxicological evidence evaluation

| Types of evidence | | A | B | C | The levels of evidence in this research |
| --- | --- | --- | --- | --- | --- |
| HIE | The research basis of the drug | The injury of toxic components is confirmed, and the difference is significant compared with the healthy control group | The drug has toxicity but its toxic component is not clear | The toxicity of the drug is not clear | A |
|  | The type of toxic components | The pure substance (monomer) | The extraction site of components upon partition and purification | The crude extract (only upon solvent extraction and the component remains obscure) | B |
| IPE | Appearance and behavior observation | The evaluation index can be quantified | The evaluation index consists of quantifiable and non-quantifiable indexes | The evaluation indexes are not quantifiable | B |
| AOE | Toxic damage detection technology at the tissue and organ level | The confirmation of the histopathological section and other parenchymal lesions | The serum biochemical indexes related to function were comprehensive | The serum biochemical indexes were not comprehensive | B |
| TEE | Intestinal microbiome | The quantitative verification of differential microbiota has been carried out | The relative abundance on microbiota has been carried out | The significant information of differential microbiota is incomplete | B |
|  | Untargeted metabolomics | Quantitative verification of differential metabolites has been carried out | Metabolites with significant differences, fold changes and high correlations have been obtained | The important information of differential metabolites is incomplete | A |
|  | Transcriptomics | Quantitative verification of differential mRNA is carried out | The significant difference, multiple changes and high correlation mRNA are obtained | The differential mRNA is not processed and screened | B |

#### The references of supplementary material

[1] Hui Gao, Xiaoli Xiong, Qing Zhang*, et al.* Analysis of composition changes of Polygalae Radix before and after processing based on UPLC-LTQ-Orbitrap MS [J]. *Traditional Chinese Drug Research and Clinical Pharmacology*, 2021, 32(12): 1845-54.

[2] Wu An-Guo, Wong Vincent Kam-Wai, Zeng Wu*, et al.* Identification of novel autophagic Radix Polygalae fraction by cell membrane chromatography and UHPLC-(Q)TOF-MS for degradation of neurodegenerative disease proteins [J]. *Scientific Reports*, 2015, 5: 17199.

[3] Lin Ruimei, Yin Jiaxin, Wu Mengfan*, et al.* Global identification and determination of the major constituents in Kai-Xin-San by ultra-performance liquid chromatography-quadrupole-Orbitrap mass spectrometry and gas chromatography-mass spectrometry [J]. *Journal of Pharmaceutical and Biomedical Analysis*, 2021, 206: 114385.

[4] Ling Yun, Li Zhixiong, Chen Mingcang*, et al.* Analysis and detection of the chemical constituents of Radix Polygalae and their metabolites in rats after oral administration by ultra high-performance liquid chromatography coupled with electrospray ionization quadrupole time-of-flight tandem mass spectrometry [J]. *Journal of Pharmaceutical and Biomedical Analysis*, 2013, 85: 1-13.

[5] Zhang Fusheng, Li Xiaowei, Li Zhenyu*, et al.* UPLC/Q-TOF MS-Based Metabolomics and qRT-PCR in Enzyme Gene Screening with Key Role in Triterpenoid Saponin Biosynthesis of Polygala tenuifolia [J]. *PLoS ONE*, 2014, 9(8): e105765.

[6] Song Yuelin, Song Qingqing, Li Jun*, et al.* Chromatographic analysis of Polygalae Radix by online hyphenating pressurized liquid extraction [J]. *Scientific Reports*, 2016, 6: 27303.

[7] Feng Gui-Fang, Liu Shu, Pi Zi-Feng*, et al.* Studies on the chemical and intestinal metabolic profiles of Polygalae Radix by using UHPLC-IT-MS and UHPLC-Q-TOF-MS method coupled with intestinal bacteria incubation model in vitro [J]. *Journal of Pharmaceutical and Biomedical Analysis*, 2018, 148: 298-306.

[8] Li J, Wang DD, Xu XS*, et al.* Utilization of UPLC/Q-TOF-MS-based metabolomics and AFLP-based marker-assisted selection to facilitate/assist conventional breeding of Polygala tenuifolia [J]. *Chemistry & biodiversity*, 2017, 14(9): e1700163.

[9] Qirun Chen, Xu Zhang. Triterpenoid constituents and pharmacological activities of Polygalae [J]. *Chinese Journal of Ethnomedicine and Ethnopharmacy*, 2019, 28(19): 49-56.

[10] Wu Dan, He Jirui, Jiang Yueming*, et al.* Quality analysis of Polygala tenuifolia root by ultrahigh performance liquid chromatography-tandem mass spectrometry and gas chromatography-mass spectrometry [J]. *Journal of Food and Drug Analysis*, 2015, 23(1): 144-51.
